# No Evidence for Genome-Wide Interactions on Plasma Fibrinogen by Smoking, Alcohol Consumption and Body Mass Index: Results from Meta-Analyses of 80,607 Subjects

**DOI:** 10.1371/journal.pone.0111156

**Published:** 2014-12-31

**Authors:** Jens Baumert, Jie Huang, Barbara McKnight, Maria Sabater-Lleal, Maristella Steri, Audrey Y. Chu, Stella Trompet, Lorna M. Lopez, Myriam Fornage, Alexander Teumer, Weihong Tang, Alicja R. Rudnicka, Anders Mälarstig, Jouke-Jan Hottenga, Maryam Kavousi, Jari Lahti, Toshiko Tanaka, Caroline Hayward, Jennifer E. Huffman, Pierre-Emmanuel Morange, Lynda M. Rose, Saonli Basu, Ann Rumley, David J. Stott, Brendan M. Buckley, Anton J. M. de Craen, Serena Sanna, Marco Masala, Reiner Biffar, Georg Homuth, Angela Silveira, Bengt Sennblad, Anuj Goel, Hugh Watkins, Martina Müller-Nurasyid, Regina Rückerl, Kent Taylor, Ming-Huei Chen, Eco J. C. de Geus, Albert Hofman, Jacqueline C. M. Witteman, Moniek P. M. de Maat, Aarno Palotie, Gail Davies, David S. Siscovick, Ivana Kolcic, Sarah H. Wild, Jaejoon Song, Wendy L. McArdle, Ian Ford, Naveed Sattar, David Schlessinger, Anne Grotevendt, Maria Grazia Franzosi, Thomas Illig, Melanie Waldenberger, Thomas Lumley, Geoffrey H. Tofler, Gonneke Willemsen, André G. Uitterlinden, Fernando Rivadeneira, Katri Räikkönen, Daniel I. Chasman, Aaron R. Folsom, Gordon D. Lowe, Rudi G. J. Westendorp, P. Eline Slagboom, Francesco Cucca, Henri Wallaschofski, Rona J. Strawbridge, Udo Seedorf, Wolfgang Koenig, Joshua C. Bis, Kenneth J. Mukamal, Jenny van Dongen, Elisabeth Widen, Oscar H. Franco, John M. Starr, Kiang Liu, Luigi Ferrucci, Ozren Polasek, James F. Wilson, Tiphaine Oudot-Mellakh, Harry Campbell, Pau Navarro, Stefania Bandinelli, Johan Eriksson, Dorret I. Boomsma, Abbas Dehghan, Robert Clarke, Anders Hamsten, Eric Boerwinkle, J. Wouter Jukema, Silvia Naitza, Paul M. Ridker, Henry Völzke, Ian J. Deary, Alexander P. Reiner, David-Alexandre Trégouët, Christopher J. O'Donnell, David P. Strachan, Annette Peters, Nicholas L. Smith

**Affiliations:** 1 Institute of Epidemiology II, Helmholtz Zentrum München, German Research Center for Environmental Health, Neuherberg, Germany; 2 National Heart, Lung and Blood Institute's Framingham Heart Study, Framingham, Massachusetts, United States of America; 3 National Heart, Lung and Blood Institute Division of Intramural Research, Bethesda, Maryland, United States of America; 4 Department of Human Genetics, Wellcome Trust Sanger Institute, Hinxton, Cambridge, United Kingdom; 5 Department of Biostatistics, University of Washington, Seattle, Washington, United States of America; 6 Cardiovascular Genetics and Genomics Group, Atherosclerosis Research Unit, Department of Medicine, Karolinska Institutet, Karolinska University Hospital Solna, Stockholm, Sweden; 7 Istituto di Ricerca Genetica e Biomedica, Consiglio Nazionale delle Ricerche, Cagliari, Italy; 8 Division of Preventive Medicine, Brigham and Women's Hospital, Boston, Massachusetts, United States of America; 9 Department of Cardiology, Leiden University Medical Center, Leiden, the Netherlands; 10 Department of Gerontology and Geriatrics, Leiden University Medical Center, Leiden, the Netherlands; 11 Department of Psychology, The University of Edinburgh, Edinburgh, United Kingdom; 12 Centre for Cognitive Ageing and Cognitive Epidemiology, The University of Edinburgh, Edinburgh, United Kingdom; 13 Brown Foundation Institute of Molecular Medicine, Division of Epidemiology, School of Public Health, University of Texas Health Science Center at Houston, Houston, Texas, United States of America; 14 Interfaculty Institute for Genetics and Functional Genomics, Ernst-Moritz-Arndt-University Greifswald, Greifswald, Germany; 15 Division of Epidemiology and Community Health, University of Minnesota, Minneapolis, Minnesota, United States of America; 16 Division of Population Health Sciences & Education, St George's, University of London, Cranmer Terrace, London, United Kingdom; 17 Department of Biological Psychology, VU University & EMGO+ institute, VU Medical Centre, Amsterdam, the Netherlands; 18 Department of Epidemiology, Erasmus Medical Center, Rotterdam, the Netherlands; 19 Netherlands Genomics Initiative (NGI)-Sponsored Netherlands Consortium for Healthy Aging (NCHA), Rotterdam, the Netherlands; 20 Institute of Behavioural Sciences, University of Helsinki, Helsinki, Finland; 21 Folkhalsan Research Centre, Helsinki, Finland; 22 Translational Gerontology Branch, National Institute on Aging, Baltimore, Maryland, United States of America; 23 MRC Human Genetics Unit, Institute of Genetics and Molecular Medicine, Western General Hospital, Edinburgh, Scotland, United Kingdom; 24 INSERM UMR_S 1062, Aix-Marseille Université, Marseille, France; 25 Division of Biostatistics, University of Minnesota, Minneapolis, Minnesota, United States of America; 26 Division of Cardiovascular and Medical Sciences, University of Glasgow, Glasgow, United Kingdom; 27 Institute of Cardiovascular and Medical Sciences, Faculty of Medicine, University of Glasgow, Glasgow, United Kingdom; 28 Department of Pharmacology and Therapeutics, University College Cork, Cork, Ireland; 29 Department of Prosthetic Dentistry, Gerostomatology and Dental Materials, University Medicine Greifswald, Greifswald, Germany; 30 Science for Life Laboratory, Karolinska Insitutet, Stockholm, Sweden; 31 Department of Cardiovascular Medicine, University of Oxford, John Radcliffe Hospital, Headington, Oxford, United Kingdom; 32 Department of Cardiovascular Medicine, The Wellcome Trust Centre for Human Genetics, University of Oxford, Oxford, United Kingdom; 33 Institute of Genetic Epidemiology, Helmholtz Zentrum München, German Research Center for Environmental Health, Neuherberg, Germany; 34 Institute of Medical Informatics, Biometry and Epidemiology, Chair of Genetic Epidemiology, Ludwig-Maximilians-Universität München, Munich, Germany; 35 Department of Medicine I, University Hospital Grosshadern, Ludwig-Maximilians-Universität München, Munich, Germany; 36 ESC-Environmental Science Center, University of Augsburg, Augsburg, Germany; 37 Medical Genetics Institute, Cedars-Sinai Medical Center, Los Angeles, California, United States of America; 38 Department of Biostatistics, Boston University, Boston, Massachusetts, United States of America; 39 Department of Haematology, Erasmus Medical Center, Rotterdam, the Netherlands; 40 Wellcome Trust Sanger Institute, Wellcome Trust Genome Campus, Cambridge, United Kingdom; 41 Institute for Molecular Medicine Finland (FIMM), University of Helsinki, Helsinki, Finland; 42 Department of Medical Genetics, University of Helsinki and University Central Hospital, Helsinki, Finland; 43 Department of Epidemiology, University of Washington, Seattle, Washington, United States of America; 44 Department of Public Health, University of Split Medical School, Split, Croatia; 45 Centre for Population Health Sciences, University of Edinburgh, Teviot Place, Edinburgh, Scotland, United Kingdom; 46 School of Social and Community Medicine, University of Bristol, Bristol, United Kingdom; 47 Robertson Center for Biostatistics, University of Glasgow, Glasgow, United Kingdom; 48 BHF Glasgow Cardiovascular Research Centre, Faculty of Medicine, Glasgow, United Kingdom; 49 Intramural Research Program, National Institute on Aging, Baltimore, Maryland, United States of America; 50 Institute for Clinical Chemistry and Laboratory Medicine, University Medicine Greifswald, Greifswald, Germany; 51 Department of Cardiovascular Research, IRCCS Istituto di Ricerche Farmacologiche Mario Negri, Milan, Italy; 52 Research Unit of Molecular Epidemiology, Helmholtz Zentrum München, German Research Center for Environmental Health, Neuherberg, Germany; 53 Hannover Unified Biobank, Hannover Medical School, Hannover, Germany; 54 Department of Statistics, University of Auckland, Auckland, New Zealand; 55 Royal North Shore Hospital, University of Sydney, Sydney, Australia; 56 Department of Internal Medicine, Erasmus Medical Center, Rotterdam, the Netherlands; 57 Division of Preventive Medicine, Division of Genetics, Brigham and Women's Hospital and Harvard Medical School, Boston, Massachusetts, United States of America; 58 Institute of Cardiovascular & Medical Sciences, University of Glasgow, Glasgow, United Kingdom; 59 Department of Molecular Epidemiology, Leiden University Medical Center, Leiden, the Netherlands; 60 DZHK (German Centre for Cardiovascular Research), partner site Greifswald, Greifswald, Germany; 61 Leibniz-Institut für Arterioskleroseforschung an der Universität Münster, Münster, Germany; 62 Department of Internal Medicine II - Cardiology, University of Ulm Medical Center, Ulm, Germany; 63 Cardiovascular Health Research Unit, Department of Medicine, University of Washington, Seattle, Washington, United States of America; 64 Harvard Medical School, Boston, Massachusetts, United States of America; 65 Division of General Medicine and Primary Care, Beth Israel Deaconess Medical Center, Boston, Massachusetts, United States of America; 66 Alzheimer Scotland Dementia Research Centre, The University of Edinburgh, Edinburgh, United Kingdom; 67 Department of Preventive Medicine, Northwestern University Medical School, Chicago, Illinois, United States of America; 68 INSERM, UMR_S 1166, Paris, France; 69 Sorbonne Universités, UPMC Univ Paris 06, UMR_S 1166, Team Genomics & Pathophysiology of Cardiovascular Diseases, Paris, France; 70 ICAN Institute for Cardiometabolism and Nutrition, Paris, France; 71 Geriatric Unit, Azienda Sanitaria Firenze (ASF), Florence, Italy; 72 National Institute for Health and Welfare, Helsinki, Finland; 73 Department of General Practice and Primary Health Care, University of Helsinki, Helsinki, Finland; 74 Helsinki University Central Hospital, Unit of General Practice, Helsinki, Finland; 75 Clinical Trial Service Unit, University of Oxford, Oxford, United Kingdom; 76 Human Genetics Center and Institute of Molecular Medicine, University of Texas Health Science Center, Houston, Texas, United States of America; 77 Durrer Center for Cardiogenetic Research, Amsterdam, the Netherlands; 78 Interuniversity Cardiology Institute of the Netherlands, Utrecht, the Netherlands; 79 Division of Preventive Medicine, Division of Cardiology, Brigham and Women's Hospital and Harvard Medical School, Boston, Massachusetts, United States of America; 80 Institute for Community Medicine, University Medicine Greifswald, Greifswald, Germany; 81 DZHK (German Centre for Cardiovascular Research), partner site Munich, Munich, Germany; 82 Group Health Research Institute, Group Health Cooperative, Seattle, Washington, United States of America; 83 Seattle Epidemiologic Research & Information Center, Veterans Affairs Office of Research & Development, Seattle, Washington, United States of America; McMaster University, Canada

## Abstract

Plasma fibrinogen is an acute phase protein playing an important role in the blood coagulation cascade having strong associations with smoking, alcohol consumption and body mass index (BMI). Genome-wide association studies (GWAS) have identified a variety of gene regions associated with elevated plasma fibrinogen concentrations. However, little is yet known about how associations between environmental factors and fibrinogen might be modified by genetic variation. Therefore, we conducted large-scale meta-analyses of genome-wide interaction studies to identify possible interactions of genetic variants and smoking status, alcohol consumption or BMI on fibrinogen concentration. The present study included 80,607 subjects of European ancestry from 22 studies. Genome-wide interaction analyses were performed separately in each study for about 2.6 million single nucleotide polymorphisms (SNPs) across the 22 autosomal chromosomes. For each SNP and risk factor, we performed a linear regression under an additive genetic model including an interaction term between SNP and risk factor. Interaction estimates were meta-analysed using a fixed-effects model. No genome-wide significant interaction with smoking status, alcohol consumption or BMI was observed in the meta-analyses. The most suggestive interaction was found for smoking and rs10519203, located in the LOC123688 region on chromosome 15, with a p value of 6.2×10^−8^. This large genome-wide interaction study including 80,607 participants found no strong evidence of interaction between genetic variants and smoking status, alcohol consumption or BMI on fibrinogen concentrations. Further studies are needed to yield deeper insight in the interplay between environmental factors and gene variants on the regulation of fibrinogen concentrations.

## Introduction

Plasma fibrinogen is an acute phase protein playing an important role in the blood coagulation cascade and is strongly associated with a variety of environmental factors such as smoking status, alcohol consumption or obesity [1]–[7]. Moreover, elevated fibrinogen concentrations indicate increased risks for developing cardiovascular diseases [8]–[10]. Genetic studies reported substantial relationships between specific genetic variants and fibrinogen concentrations [11]–[17]; the heritability of plasma fibrinogen concentrations has been estimated to range from 34% to 51% [11], [12], [18], [19]. Therefore, the regulation of fibrinogen concentrations might be seen as a complex interplay between environmental and genetic factors [20]. However, knowledge about potential interactions between environmental factors such as cardiovascular risk factors and gene variants on fibrinogen is still limited.

Smoking status, alcohol consumption and body mass index (BMI) are strong determinants of fibrinogen concentrations [1]–[7]. A large meta-analysis showed elevated fibrinogen concentrations in current smokers compared with non-smokers; moreover, fibrinogen concentrations increased with the number of cigarettes smoked per day showing a dose-related trend [7]. Similarly, elevated fibrinogen concentrations have been reported in subjects with higher BMI values [7]. For alcohol consumption, a lower mean fibrinogen concentration was observed in subjects reporting current alcohol intake compared with subjects reporting no alcohol intake [7]. All three factors represent behavioral risk factors which are easy to assess and for which we hypothesized that gene-environment interactions may modify individual risks enabling potentially targeted and individualized preventive approaches.

Several family-based and genome-wide studies have reported associations of specific gene regions with fibrinogen concentrations [11]–[17]. Recently, three large genome-wide association studies (GWAS) including as many as 90,000 participants of European origin identified up to 24 strong association signals with plasma fibrinogen concentration, among them one located in the fibrinogen *β* chain (*FGB*) gene [15]–[17]. One of these analyses [17] was essentially based on the same study population as in the present analyses.

However, little is known about whether the impact of smoking, alcohol consumption, and BMI on fibrinogen concentrations is modified by specific gene variants. Knowledge of such interactions might improve the understanding of the underlying mechanism of fibrinogen synthesis and its regulation. Two candidate-gene studies showed modifications in the association of smoking status and fibrinogen concentration by the G/A-455 polymorphism (rs1800790) located at the *FGB* gene [21], [22].

No genome-wide studies of effect modifications have been reported thus far for fibrinogen concentrations. Therefore, the aim of the present study was to assess potential gene-environment interactions (GxE) by smoking status, alcohol consumption, and BMI on fibrinogen concentrations, using genome-wide data from 22 studies with 80,607 subjects of European origin.

## Methods

### Study population

The present study was carried out within the framework of the *Cohorts for Heart and Aging Research in Genomic Epidemiology* (CHARGE) consortium which combines data from several studies of participants of European origin conducted in the United States and Europe [23]. Twenty-two studies comprising 80,607 participants provided results from their genome-wide interaction analyses for the present investigation; an overview of these studies with basic information and respective references is given in the online supporting information (S1 Methods and S1 Table) All participants provided informed consent to use their DNA for these analyses and all studies.

### Fibrinogen measurements

Plasma fibrinogen concentrations were measured in 17 studies by a functional method based on the Clauss assay and in five studies by an immunonephelometric method and given in g/L [24], [25]. Fibrinogen concentrations were approximately normally distributed in all studies and therefore analysed untransformed. More details are given in the online supporting information (S1 Methods and S1 Table).

### Assessment of smoking status, alcohol consumption and BMI

In all studies, smoking status and alcohol consumption were assessed by self-reports from study participants; assessment of BMI was based on clinical examination or self-report and described in kg/m^2^. For smoking status, current smokers (“smokers”) were compared with a combined group of former smokers and never-smokers (“non-smokers”). Former smokers were defined as not smoking at time of examination in 19 studies; the remaining 3 studies set a minimum cessation time before the examination which ranged between 30 days and 3 years. For alcohol consumption, coding was “0” for no alcohol consumption, “1” for alcohol consumption with less than 1 drink daily equivalent to less than 10 g alcohol per day and “2” for alcohol consumption with 1 or more drinks daily equivalent to 10 g alcohol or more per day. Information on smoking status and BMI was available in all 22 studies and on alcohol consumption in 20 studies. The assessments of smoking status, alcohol consumption and BMI were made at the same time as the fibrinogen measurements for all participants in all studies.

### Genotyping and imputation

Genotyping was conducted separately in each study using Affymetrix or Illumina platforms and included from ∼300,000 to ∼1,000,000 genotyped single nucleotide polymorphisms (SNPs). Genotype quality control and data cleaning based on individual call rate, SNP call rate, and/or Hardy-Weinberg equilibrium thresholds, and performed independently by each study.

Genotyped data were imputed in each study separately to the ∼2.6 million SNPs identified in the HapMap II Caucasian (CEU) sample from the Centre d'Etude du Polymorphisme Humain [26], [27].

More details about genotyping and imputation are given in the online supporting information (S1 Methods and S2 Table).

### Statistical analysis

#### Association of smoking status, alcohol consumption and BMI with fibrinogen concentration

Associations of smoking status, alcohol consumption and BMI with fibrinogen concentrations were assessed independently and separately in each study using linear regression. Analyses were adjusted for age (linear) and sex as well as study-specific covariates if required (see S1 Methods). Study-specific associations of smoking status, alcohol consumption and BMI with fibrinogen concentration were then meta-analysed using an inverse-variance weighted fixed-effect model.

#### Interaction between gene variants and smoking status, alcohol consumption and BMI on fibrinogen concentration

Genome-wide analyses of the interaction between gene variants and smoking status, alcohol consumption or BMI on fibrinogen concentration were performed independently and separately in each study assuming an additive-genetic model with an additive interaction. In all studies of unrelated individuals, linear regression models with fibrinogen concentration as the outcome variable and the SNP, the risk factor under consideration, and the interaction term ‘SNP x risk factor’ were fit with adjustments for age (linear) and sex as well as study-specific covariates if required (see S1 Methods). In family-based studies, linear mixed effects models were applied to account for family correlations. Estimates of study-specific genome-wide interactions were then meta-analysed applying inverse-variance weighted fixed-effect models. To account for population stratification, study-specific test statistics were corrected using the method of genomic control [28]. SNPs with a low minor allele frequency (MAF) (<0.05) and a low imputation quality (observed to expected variance ratio <0.3) were omitted from the meta-analyses.

The meta-analyses were performed using the software METAL developed for genome-wide data [29]. To assess heterogeneity, the I^2^ index was computed for each interaction estimate assuming that an I^2^ index around 25% or below indicates no or low and around 50% moderate heterogeneity as suggested by Higgins et al [30]. Genome-wide significance of interaction was defined as a p value <5.0×10^−8^ for each of the three GxE analyses. Power analyses were conducted using the R (version 3.0.2) *pwr* package.

## Results

### Description of studies

The study population comprised a total of 80,607 participants of European ancestry from 22 studies. Distributions of basic characteristics are provided for each study in Table 1. Across the 22 studies, the average age ranged from 43.3 to 79.1 years and the percent of male participants ranged from 0 to 75.5%. Current smoking behaviour was reported for 6.9 to 50.3% of the participants. No alcohol consumption was reported for 5.8 to 54.9% of the participants and the mean BMI ranged from 24.3 to 28.4 kg/m^2^. Mean fibrinogen concentration varied from 2.67 to 3.88 g/L.

**Table 1:** Characteristics at the time of fibrinogen measurement by study.

Study	Sample (N)	Age* (years)	Male sex (%)	Current smokers (%)	No alcohol consumption (%)	Alcohol consumption <10 g/day (%)	Alcohol consumption ≥10 g/day (%)	BMI (kg/m^2^)	Fibrinogen* (g/L)
ARIC	9,256	54.3 (5.7)	47.1	24.6	55.9	23.0	21.1	27.0 (4.8)	2.97 (0.61)
B58C	6,085	45.2 (0.4)	49.7	23.5	20.3	39.1	40.6	27.4 (4.9)	2.95 (0.60)
CARDIA	1,435	45.8 (3.3)	47.0	20.3	NA	NA	NA	25.4 (5.1)	3.18 (0.66)
CHS	3,242	72.3 (5.4)	39.0	11.3	46.0	38.5	15.5	26.3 (4.4)	3.15 (0.62)
CROATIA-Vis	761	56.6 (15.5)	41.7	27.9	43.0	19.2	37.8	27.1 (4.9)	3.58 (0.82)
FHS	2,797	54.1 (9.7)	45.5	18.5	29.8	57.9	12.3	27.4 (5.0)	3.05 (0.57)
HBCS	1,728	61.4 (2.9)	40.2	23.9	16.5	54.6	28.9	27.4 (4.5)	3.23 (1.04)
InCHIANTI	1,128	67.7 (15.1)	44.9	19.0	24.5	30.0	45.5	27.2 (4.1)	3.48 (0.75)
KORA F3	1,520	52.1 (10.2)	49.3	18.0	29.9	23.6	46.1	27.2 (4.1)	2.89 (0.66)
KORA F4	1,777	53.9 (8.9)	48.9	20.0	24.5	29.9	45.6	27.7 (4.6)	2.67 (0.60)
LBC1921	466	79.1 (0.6)	42.1	6.9	23.2	56.9	20.0	26.2 (4.1)	3.56 (0.85)
LBC1936	989	69.6 (0.8)	50.8	12.6	19.2	40.7	40.0	27.8 (4.4)	3.27 (0.63)
MARTHA	613	44.1 (14.2)	23.8	25.9	NA	NA	NA	24.3 (4.4)	3.36 (0.68)
NTR	2,343	47.1 (13.9)	35.8	16.8	5.8	74.2	20.0	25.4 (4.0)	2.78 (0.66)
ORCADES	686	53.7 (15.3)	46.6	8.7	10.8	59.2	30.0	27.7 (4.9)	3.45 (0.81)
PROCARDIS-CL	3,490	61.9 (7.0)	75.5	50.3	37.3	33.7	29.0	28.4 (4.4)	3.88 (0.86)
PROCARDIS-Im	3,405	58.1 (8.9)	73.5	33.0	23.9	36.7	39.4	27.1 (4.2)	3.86 (1.00)
PROSPER	5,244	75.3 (3.3)	48.1	26.5	44.5	28.6	27.0	26.8 (4.2)	3.60 (0.74)
RS	2,068	70.4 (9.0)	35.0	23.5	18.1	51.6	30.3	26.4 (3.8)	2.81 (0.68)
SardiNIA	4,691	43.3 (17.6)	43.7	19.8	54.9	10.2	34.9	25.3 (4.7)	3.28 (0.66)
SHIP	3,807	48.7 (16.0)	48.4	31.5	34.3	19.1	46.6	27.2 (4.8)	2.98 (0.69)
WGHS	23,076	54.7 (7.1)	0	13.2	43.3	42.7	14.0	25.9 (5.0)	3.59 (0.78)
total	80,607								

BMI: body mass index.

* Mean (standard deviation).

### Association of smoking status, alcohol consumption and BMI with fibrinogen concentration

**Figure 1:**
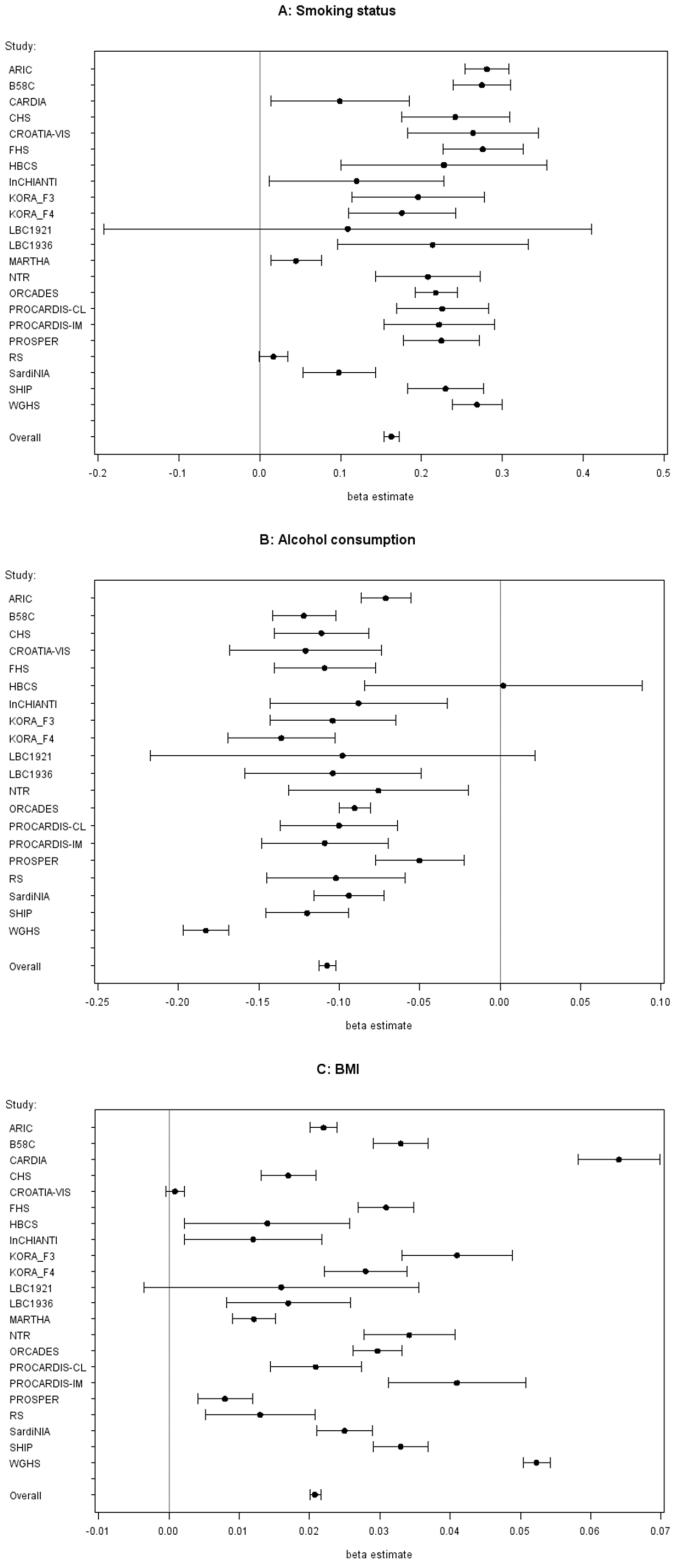
Association of environmental factors with fibrinogen concentration (in g/L), adjusted for age and sex. **A**) Forest plot for smoking status. The beta estimate with 95% confidence intervals indicates the change in mean fibrinogen concentration (in g/L) by smoking status for each study and across all studies (“overall”, estimated by meta-analysis). **B**) Forest plot for alcohol consumption. The beta estimate with 95% confidence intervals indicates the change in mean fibrinogen concentration (in g/L) by alcohol consumption for each study and across all studies (“overall”, estimated by meta-analysis). Alcohol consumption was assessed only in 20 studies. **C**) Forest plot for BMI. The beta estimate with 95% confidence intervals indicates the change in mean fibrinogen concentration (in g/L) by BMI for each study and across all studies (“overall”, estimated by meta-analysis).

Strong significant associations of smoking status, alcohol consumption and BMI with mean fibrinogen concentrations were observed in the vast majority of the 22 studies as can be seen in Fig. 1. Meta-analysis revealed mean differences in fibrinogen concentrations of 0.163 g/L (95% CI 0.154 to 0.172, 8.5×10^−280^) for current smokers compared with non-smokers, of -0.108 g/L (95% CI −0.113 to −0.102, 1.2×10^−334^) for one category increase of alcohol consumption (no, <10 g/day, ≥10 g/day), and of 0.021 g/L (95% CI 0.020 to 0.022, p value 7.1×10^−691^) for one kg/m^2^ of BMI increase.

### Interaction between gene variants and smoking status, alcohol consumption and BMI on fibrinogen concentration – genome-wide analyses

#### Overall

**Figure 2:**
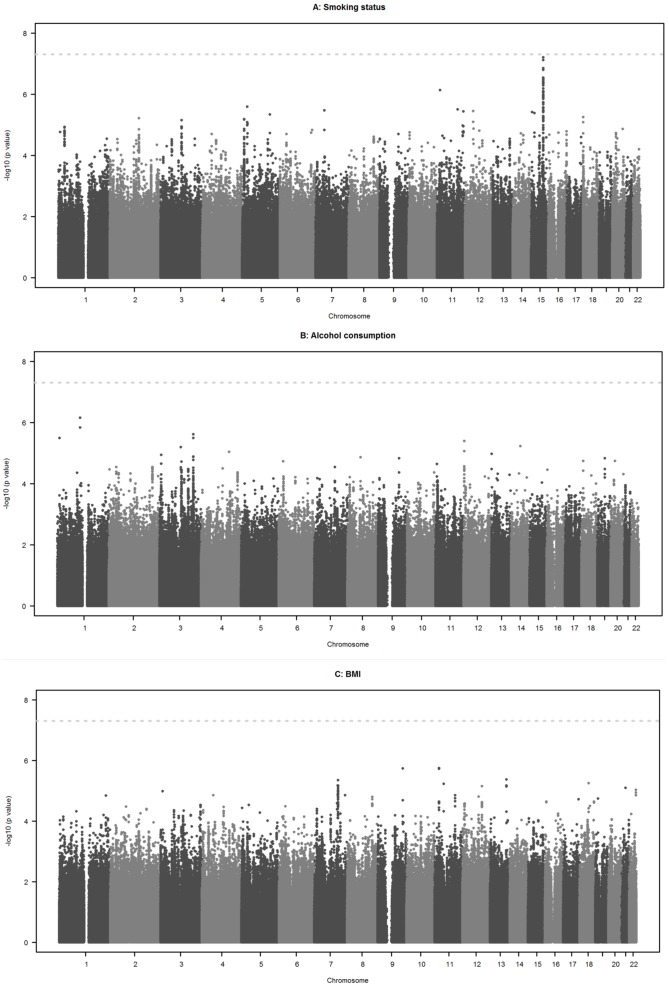
Interaction of gene variants and environmental factors on fibrinogen concentration (in g/L), adjusted for age and sex. **A**) Manhattan plot for smoking status. The horizontal axis denotes chromosome and position of each gene variant and the vertical axis gives the negative log10 of the p value for interaction of each gene variant and smoking status on fibrinogen concentration (in g/L), estimated by meta-analyses. The dotted line denotes genome-wide significance (5.0×10^−8^). **B**) Manhattan plot for alcohol consumption. The horizontal axis denotes chromosome and position of each gene variant and the vertical axis gives the negative log10 of the p value for interaction of each gene variant and alcohol consumption on fibrinogen concentration (in g/L), estimated by meta-analyses. The dotted line denotes genome-wide significance (5.0×10^−8^). Alcohol consumption was assessed only in 20 studies. **C**) Manhattan plot for BMI. The horizontal axis denotes chromosome and position of each gene variant and the vertical axis gives the negative log10 of the p value for interaction of each gene variant and BMI on fibrinogen concentration (in g/L), estimated by meta-analyses. The dotted line denotes genome-wide significance (5.0×10^−8^).

No genome-wide significant interactions with smoking status, alcohol consumption and BMI were observed on fibrinogen concentration (Fig. 2). The overall genomic inflation factors from the meta-analyses were 1.0174 for interaction with smoking status, 0.9838 for interaction with alcohol consumption and 1.0075 for interaction with BMI (see QQ plots in S1 Fig.). Exclusion of studies with a genomic inflation factor >1.15 or <1/1.15 (see S3 Table) did not substantially alter these findings and revealed no genome-wide significant interactions either. The heterogeneity of interaction estimates across studies was rather weak. More than 85% of SNPs had an I^2^ index of 25% or less in all three interaction analyses. The upper quartile of the I^2^ index value distribution was 15.5% for smoking, 16.1% for alcohol consumption and 15.5% for BMI analyses.

#### High-signal interactions

The Manhattan plot in Fig. 2 for smoking status (A) shows a peak on chromosome 15 revealing suggestive evidence of an interaction with smoking status for rs10519203, located in the *LOC123688* region on chromosome 15, with a p value of 6.2×10^−8^ (Table 2). The difference in mean fibrinogen concentration between smokers and non-smokers was 0.048 g/L lower per copy of the A allele of rs10519203. A forest plot providing the interaction estimates with smoking status for each study and for the meta-analysis is given in S2 Fig. Heterogeneity was estimated as I^2^ index = 45.4% indicating a moderate level of variation of the interaction estimates across studies. The SNP rs10519203 alone was not significantly associated with fibrinogen concentration in a “G” alone analysis (p value 0.0005) performed by Sabater-Lleal et al. [17].

**Table 2:** Interaction between gene variants and smoking status, alcohol consumption and BMI on fibrinogen concentration (in g/L) with lowest p value for interaction.

Environmental factor x SNP	Chr	Position	A1*	A2	% A1	Beta (SE)	P value	N studies	Direction**	I^2^ index
Smoking status x rs10519203	15	76601101	A	G	64.8	−0.048 (0.009)	6.2×10^−08^	22	---+—+-+-------+-+---	45.4%
Alcohol consumption x rs11102001	1	110011733	A	G	59.0	0.057 (0.012)	7.0×10^−07^	18	++++-++?+++-+++++?++	0.0%
BMI x rs7120820	11	21350435	T	C	37.6	0.004 (0.009)	1.8×10^−06^	21	++++-++—+-+-+---++?++	20.2%

BMI: body mass index, SNP: single nucleotide polymorphism, Chr: chromosome, SE: standard error.

* Allele 1 is effect allele, ** The order of studies under “direction” refers to the order of studies in *Table 1* *.*

For alcohol consumption and BMI, the lowest p values were found for rs11102001 in *EPS8L3* on chromosome 1 (p value = 7.0×10^−7^) and rs7120820 in *NELL1* on chromosome 11 (p value = 1.8×10^−6^), see Table 2. In both cases, weak heterogeneity was observed (I^2^ index≤25%).

### Interaction between gene variants and smoking status, alcohol consumption and BMI on fibrinogen concentration - selected candidate SNPs

The interaction of rs1800790 at the *FGB* locus and smoking status on fibrinogen concentration (as it was shown in two candidate-gene approach studies previously) was estimated with beta  = 0.029 in the present meta-analysis (p value for interaction 0.006) indicating a 0.029 g/L higher level in mean fibrinogen concentration per A allele copy in smokers compared with non-smokers (Table 3). The I^2^ index of almost 60% indicated moderate heterogeneity.

**Table 3:** Interaction with smoking status for association with fibrinogen concentration (in g/L) in SNPs with previously reported interactions (Green et al. 1993; Thomas et al. 1996) or among SNPs associated with circulating fibrinogen (Sabater-Leal et al. 2013).

SNP	Chr	Position	A1*	A2	% A1	Beta (SE)	P value	N studies	Direction**	I^2^ index
Significant interaction according to Green et al. (1993) and Thomas et al. (1996):										
rs1800790	4	155703158	A	G	23.2	0.029 (0.011)	0.0062	22	+++++---++-+++-++—+—	59.2%
Significant SNPs according to Sabater-Leal et al. (2013):										
rs1938492	1	65890417	A	C	62.1	0.017 (0.009)	0.058	22	++-+---+-+++-+-+++—++	0.0%
rs4129267	1	152692888	T	C	39.1	−0.004 (0.009)	0.614	22	+-+-+-++—+++++---++-+	43.9%
rs10157379	1	245672222	T	C	62.3	0.008 (0.009)	0.386	21	------+++++-++++++-?++	0.0%
rs12712127	2	102093093	A	G	40.6	0.009 (0.009)	0.286	22	++-++-++—+-++-+++++—	0.0%
rs6734238	2	113557501	A	G	58.6	−0.005 (0.009)	0.561	22	+---+++-+-+-+—+-+-+-+	0.8%
rs715	2	211251300	T	C	68.0	−0.007 (0.011)	0.501	17	-?+?-+—?+—?+-+—+?+-	0.7%
rs1476698	2	241945122	A	G	64.5	0.008 (0.009)	0.357	22	++------+---+—++++-++	34.9%
rs1154988	3	137407881	A	T	78.1	−0.013 (0.010)	0.194	22	-----+-++++---+—+-+-+	0.0%
rs16844401	4	3419450	A	G	7.5	0.017 (0.020)	0.392	18	—??+?-+++++—+++-+?-+	1.0%
rs1800789	4	155702193	A	G	21.1	0.031 (0.011)	0.0028	22	+++++---++-++—++-----	61.1%
rs11242111	5	131783957	A	G	5.7	0.051 (0.033)	0.124	9	+??+?+?+?-?++?-????+??	30.0%
rs2106854	5	131797073	T	C	20.8	−0.004 (0.011)	0.737	22	---------+-+++++-+++++	0.0%
rs10226084	7	17964137	T	C	51.9	−0.008 (0.009)	0.374	22	-++++---+---+-++---+—	0.0%
rs2286503	7	22823131	T	C	36.1	−0.013 (0.009)	0.131	22	+-+-++++++—+-+----+—	0.0%
rs7464572	8	145093155	C	G	59.7	−0.004 (0.009)	0.672	19	+?++----?-++?-+----+-+	0.0%
rs7896783	10	64832159	A	G	48.4	−0.014 (0.008)	0.094	22	-+-++—++-----+----+—	0.0%
rs1019670	11	59697175	A	T	35.8	−0.009 (0.009)	0.345	22	+---++++++---+----+---	0.0%
rs7968440	12	49421008	A	G	64.0	0.003 (0.009)	0.855	22	+-+-+----+----++++—++	52.0%
rs434943	14	68383812	A	G	31.7	−0.002 (0.010)	0.884	21	-----+-++++----++++?++	0.0%
rs12915708	15	48835894	C	G	30.6	−0.003 (0.009)	0.776	22	-+—++---+-++----+-+—	20.7%
rs7204230	16	51749832	T	C	69.7	0.008 (0.010)	0.447	19	+?-++—+?+—?-+++++-++	0.0%
rs10512597	17	70211428	T	C	17.9	−0.008 (0.012)	0.490	21	----+-+—+-+—+-+++?++	0.0%
rs4817986	21	39387382	T	G	27.9	−0.025 (0.010)	0.011	20	—?+---+-----++---+?—	12.8%
rs6010044	22	49448804	A	C	79.5	0.005 (0.012)	0.669	21	+—++++---++++—?-++++	0.0%

SNP: single nucleotide polymorphism, Chr: chromosome, SE: standard error.

* Allele 1 is effect allele, ** The order of studies under “direction” refers to the order of studies in *Table 1* *.*

Finally, we examined 24 signals previously shown to be associated with circulating fibrinogen in genome-wide analysis in almost the same study population [17] assuming a significance threshold of 0.0021 (Bonferroni correction for 24 tests: 0.05/24). None of these candidates was significant at this threshold in the present GxE meta-analyses. For smoking status, a suggestive interaction was found for rs1800789 in the *FGB* gene on chromosome 4 (p value for interaction 0.0028) revealing a 0.031 g/L higher difference in mean fibrinogen concentration per A allele copy in smokers compared with non-smokers (Table 3). This SNP represents almost the same signal as rs1800790 (r^2^ = 0.911, D′ = 1.00) for which interactions with smoking was found previously and a similar I^2^ index was estimated (see above).

For alcohol consumption and BMI, the lowest p values among the 24 signals were observed for rs715 in *CPS1* on chromosome 2 (p value = 0.0195) and for rs10512597 in *CD300LF* on chromosome 17 (p value = 0.0049), see S4 and S5 Tables.

## Discussion

### Overall

The present study is the first to investigate interactions between smoking status, alcohol consumption, and BMI and gene variants on fibrinogen concentrations based on data from genome-wide interaction studies. Meta-analysing a population of 80,607 participants of European ancestry drawn from 22 studies did not identify any variant that modified the association of smoking status, alcohol consumption and BMI with plasma fibrinogen concentrations with genome-wide significance.

### Association of smoking status, alcohol consumption and BMI with fibrinogen concentration

Several studies have identified strong and significant associations of smoking status, alcohol consumption and BMI with fibrinogen concentrations [1]–[7]. The present study confirmed these associations and is in line with findings from a large meta-analysis of 154,211 participants in 31 prospective studies conducted by the Fibrinogen Studies Collaboration (FSC) which showed comparable estimates of fibrinogen concentration differences in smokers compared to non-smokers and for differences in BMI and alcohol consumption amounts [7]. These strong associations of smoking status, alcohol consumption and BMI with fibrinogen might be explained mainly by their relation with the acute phase reaction which contributes to the regulation of the fibrinogen synthesis [1].

### Interaction between gene variants and smoking status, alcohol consumption and BMI on fibrinogen concentration – genome-wide analyses

The present study found no evidence that the strong associations of smoking status, alcohol consumption and BMI with fibrinogen concentrations were significantly modified by any of the approximately 2.6 million polymorphisms identified in the HapMap II Caucasian (CEU) sample. However, a peak on chromosome 15 with suggestive evidence of an interaction with smoking status was found for rs10519203 (p value for interaction 6.2×10^−8^) which is located in the *LOC123688* region. The effect on fibrinogen level by smoking status was lower per copy of the A allele (major allele) indicating that fibrinogen regulation by tobacco exposure might be attenuated depending on the genotype of rs10519203. A strong association of genetic variants in the *LOC123688* region with lung cancer has been reported recently [31]–[33]. One of these variants, rs8034191, was in complete linkage disequilibrium with rs10519203 (r^2^ = 1, D′ = 1). Interestingly, Truong et al. found that these variants were associated with significantly increased lung cancer risk per copy of their minor allele in former or current but not in never smokers [33].

### Interaction between gene variants and smoking status, alcohol consumption and BMI on fibrinogen concentration - selected candidate SNPs

Two previous studies with a candidate-gene approach reported interactions between the G/A-455 polymorphism at the *FGB* gene (rs1800790) and smoking status on fibrinogen concentration in samples of healthy men; however, these studies reported contradictory findings [21], [22]: whereas, in 86 healthy men, fibrinogen concentrations were significantly higher per copy of the A allele in smokers only [21], the opposite was true in 482 healthy middle-aged men with significant associations only in non-smokers [22]. The present meta-analyses could confirm the findings of an association only in smokers [21] albeit with a sample size more than 200 times larger.

Several studies have identified strong associations of specific variants with fibrinogen levels; one of these was in the fibrinogen *β* chain *(FBG)* gene [15]–[17]. A very recently performed meta-analysis conducted also within the framework of the CHARGE consortium and comprising almost the same study population as the present investigation revealed 24 independent signals in 23 loci being significantly associated with fibrinogen concentration [17]. The present GxE meta-analyses indicated no significant modifications of the associations of smoking status, alcohol consumption and BMI with fibrinogen concentration.

### Strengths and limitations

The present study was restricted to genetic variants with a minor allele frequency of at least 5%. Analyses for variants with a lower MAF produced an excess of small p values which were likely due to a poor approximation of true null distribution of the test statistics by the normal distribution. It is possible, however, that there may be significant interactions for rare variants which could be detected in studies with improved approximations or with even larger sample sizes. Moreover, the three determinants of fibrinogen were employed in commonly used categorizations across all studies; however, other definitions (e.g. never smokers versus ever smokers, other cut-off values than 10 g/day for alcohol consumption or categorized BMI) might yield significant interactions. Finally, we observed heterogeneity in covariate distribution and effect estimates which may affect our findings.

The present study is the first genome-wide interaction study aimed to detect interactions between environmental factors and gene variants on fibrinogen concentration. Its strength relies on the large numbers of studies and participants which was confirmed by a power analysis: If we assume a study population with a prevalence of exposed of ≥20% (e.g. smokers), a SNP with a MAF of ≥0.05, and an interaction of ≥0.1 meaning that the difference in mean fibrinogen concentration between exposed and non-exposed participants is at least 0.1 g/L higher per one copy of the minor allele, the power to detect an interaction would be greater than 90% based on 80,000 participants and a significance level of 5.0×10^−8^.These estimations indicate that the power of our study is large enough to detect genome-wide relevant interactions between SNPs and smoking, alcohol consumption and BMI on fibrinogen concentration.

### Conclusions

The present large genome-wide interaction analyses including 22 studies comprising 80,607 subjects of European ancestry did not identify significant interaction of gene variants and smoking status, alcohol consumption or BMI on fibrinogen concentrations. The strong associations of these three variables with fibrinogen are not modified substantially by any of the 2.6 million common genetic variants analysed in this study. Suggestive evidence of an interaction could be found for smoking status with a fibrinogen-SNP association in smokers and but not in non-smokers. Further studies are needed to yield deeper insight in the interplay between environmental factors and functional genomics on the regulation of fibrinogen concentrations.

## Supporting Information

S1 Fig QQ plots for interaction of gene variants and environmental factors on fibrinogen concentration (in g/L), adjusted for age and sex.
(TIF)

S2 Fig Forest plot for interaction of rs10519203 and smoking status on fibrinogen concentration (in g/L) with 95% confidence interval, adjusted for age and sex.
(TIF)

S1 Table **Basic information about studies.**
(DOC)

S2 Table **Genotype information about studies.**
(DOC)

S3 Table **Genomic inflation factor per study.**
(DOC)

S4 Table **Interaction with alcohol consumption for association with fibrinogen concentration (in g/L) among SNPs associated with circulating fibrinogen (Sabater-Leal et al. 2013).**
(DOC)

S5 Table **Interaction with BMI for association with fibrinogen concentration (in g/L) among SNPs associated with circulating fibrinogen (Sabater-Leal et al. 2013).**
(DOC)

S1 Funding **Funding statement.**
(DOC)

S1 Checklist **PRISMA checklist.**
(DOC)

S1 Methods **This file gives additional information about the study samples, fibrinogen measurements, genotyping/imputation and statistical analyses.**
(DOC)
