# Supplementary figures and images for: No Evidence for Genome-Wide Interactions on Plasma Fibrinogen by Smoking, Alcohol Consumption and Body Mass Index: Results from Meta-Analyses of 80,607 Subjects
Source: PLoS One. 2014 Dec 31;9(12):e111156. doi: 10.1371/journal.pone.0111156 (PMC4281156; doi:10.1371/journal.pone.0111156)

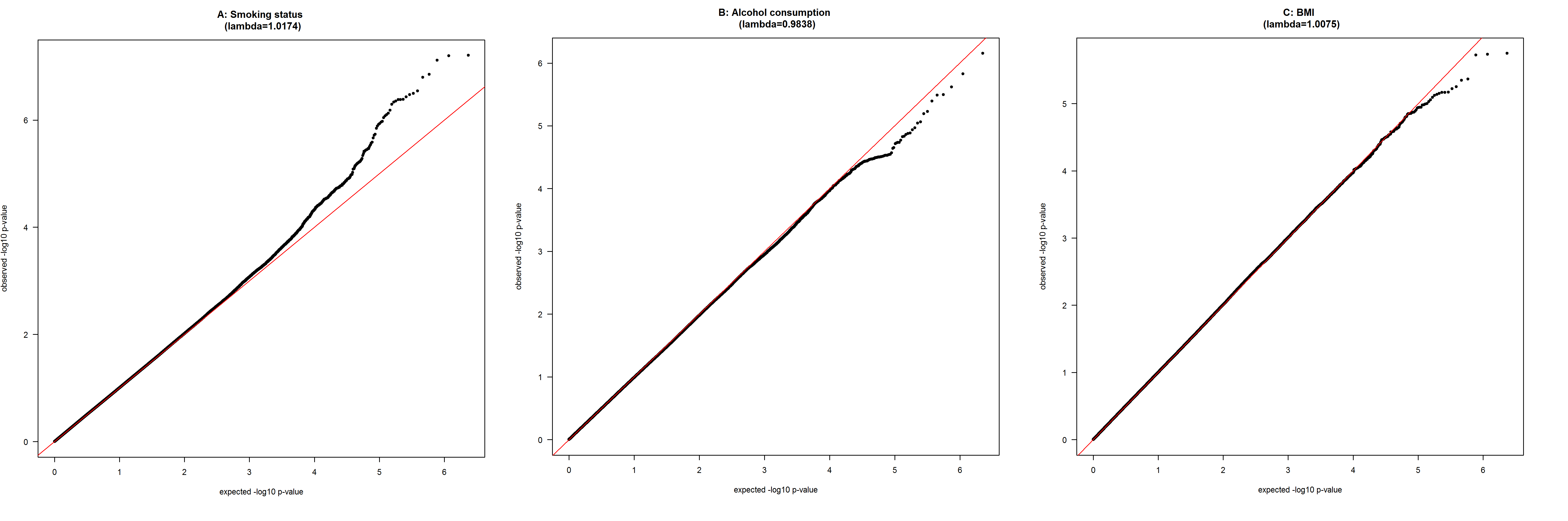

Supplement: S1 Fig — QQ plots for interaction of gene variants and environmental factors on fibrinogen concentration (in g/L), adjusted for age and sex. (TIF) [file pone.0111156.s001.tif]

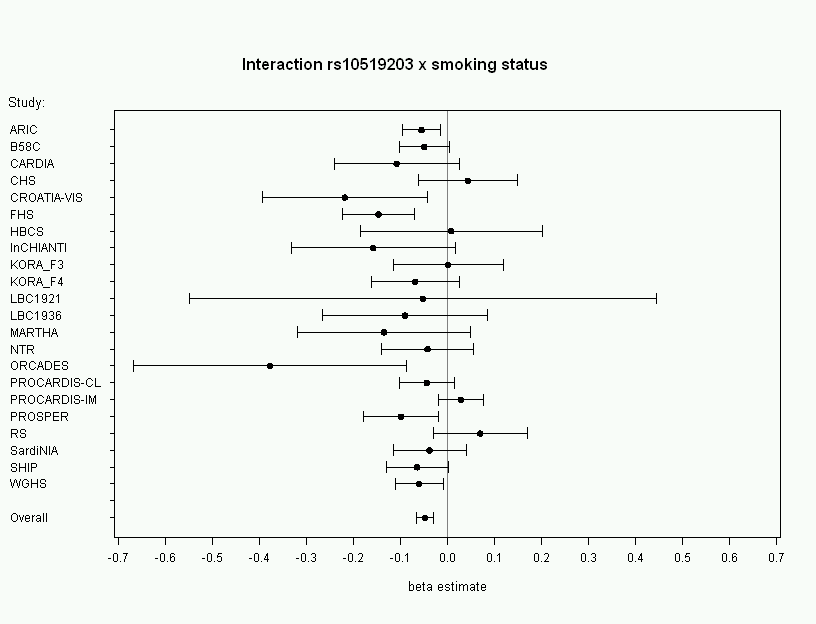

Supplement: S2 Fig — Forest plot for interaction of rs10519203 and smoking status on fibrinogen concentration (in g/L) with 95% confidence interval, adjusted for age and sex. (TIF) [file pone.0111156.s002.tif]
