# Supplementary material for: No Evidence for Genome-Wide Interactions on Plasma Fibrinogen by Smoking, Alcohol Consumption and Body Mass Index: Results from Meta-Analyses of 80,607 Subjects
Source: PLoS One. 2014 Dec 31;9(12):e111156. doi: 10.1371/journal.pone.0111156 (PMC4281156; doi:10.1371/journal.pone.0111156)
Supplement: S1 Table — Basic information about studies. (DOC) [file pone.0111156.s003.doc]

**Table S1.** Basic information about studies.

| **Study** | **Sample (N)** | **Study design** | **European ancestry** | **Age range**  **(in years)** | **Fibrinogen**  **assay** | **Fibrinogen measurement** |
| --- | --- | --- | --- | --- | --- | --- |
| ARIC | 9,256 | community-based | 100% | 45-64 | Clauss | Citrate/EDTA |
| B58C | 6,085 | national birth cohort | 100% | 44-45 | Clauss | Citrate |
| CARDIA | 1,435 | community-based | 100% | 37-52 | nephelometric | Citrate/EDTA |
| CHS | 3,242 | community-based | 100% | 65-98 | Clauss | Citrate |
| CROATIA-Vis | 761 | population-based | 100% | 18-93 | Clauss | Citrate |
| FHS | 2,797 | community-based | 100% | 26-82 | Clauss | Citrate |
| HBCS | 1,728 | birth cohort | 100% | 56-69 | Clauss | EDTA |
| InCHIANTI | 1,128 | population-based | 100% | 23-95 | Clauss | Citrate |
| KORA F3 | 1,520 | population-based | 100% | 25-69 | nephelometric | EDTA |
| KORA F4 | 1,777 | population-based | 100% | 25-74 | nephelometric | EDTA |
| LBC1921 | 466 | birth cohort | 100% | 78-81 | Clauss | Citrate |
| LBC1936 | 989 | birth cohort | 100% | 68-71 | Clauss | Citrate |
| MARTHA | 613 | VT patients | 100% | 1-91 | Clauss | Citrate |
| NTR | 2,343 | population-based | 100% | 18-90 | Clauss | Citrate |
| ORCADES | 686 | population-based | 100% | 18-100 | Clauss | Citrate |
| PROCARDIS-CL | 3,490 | CAD cases | 100% | 35-86 | Clauss | Citrate |
| PROCARDIS-Im | 3,405 | CAD cases + controls | 100% | 20-84 | nephelometric | EDTA |
| PROSPER | 5,244 | prospective randomized controlled trial | 100% | 70-82 | Clauss | EDTA |
| RS | 2,068 | population-based | 100% | 55-99 | Functional* | Citrate |
| SardiNIA | 4,691 | population-based | 100% | 14-102 | Clauss | Citrate |
| SHIP | 3,807 | population-based | 100% | 20-81 | Clauss | Citrate |
| WGHS | 23,076 | population-based | 100% | 38-90 | immunoturbidimetric | Citrate/EDTA |
| Total | 80,607 |  |  |  |  |  |

** Functional PT-derived method (Rossi E, Mondonico P, Lombardi A, Preda L. Method for the determination of functional (clottable) fibrinogen by the new family of ACL coagulometers. Thromb Res. 1988; 52:453-68).*
