# Supplementary material for: No Evidence for Genome-Wide Interactions on Plasma Fibrinogen by Smoking, Alcohol Consumption and Body Mass Index: Results from Meta-Analyses of 80,607 Subjects
Source: PLoS One. 2014 Dec 31;9(12):e111156. doi: 10.1371/journal.pone.0111156 (PMC4281156; doi:10.1371/journal.pone.0111156)
Supplement: S2 Table — Genotype information about studies. (DOC) [file pone.0111156.s004.doc]

**Table S2.** Genotype information about studies.

| **Study** | **Genotyping plattform** | **Calling algorithm** | **NCBI build** | **Imputation software** | **Analysis software** |
| --- | --- | --- | --- | --- | --- |
| ARIC | Affymetrix 6.0 | Birdseed | 36 | MACH  v1.0.16 | ProbABEL |
| B58C | Illumina 550K or 610K (3 deposits) | BeadStudio (GenCall) | 35.21 | MACH v1.0.16 | ProbABEL |
| CARDIA | Affymetrix 6.0 | Birdseed + BEAGLE | 36.22 | BEAGLE | ProbABEL |
| CHS | Illumina 370CNV | Illumina BeadStudio | 36 | BIMBAM  v0.99 | R |
| CROATIA-Vis | Illumina HumanHap300 v1 | Illumina | 36.22 | MACH v1.0.16 | ProbABEL |
| FHS | Affymetrix 500K + 50K | BLRMM | 36.2 | MACH v1.0.15 | R* |
| HBCS | modified Illumina Human 610_Quad | Illumina | 36.2 | MACH | Quicktest,  ProbABEL |
| InCHIANTI | Illumina 550K | Beadstudio | 36 | MACH  v1.0 16 | ProbABEL |
| KORA F3 | Affymetrix 500K | BLRMM | 35.21 | MACH v1.0.9 | ProbABEL |
| KORA F4 | Affymetrix 6.0 (1000K) | Birdseed2 | 36.22 | MACH v1.0.15 | ProbABEL |
| LBC1921 | Illumina Human 610_Quadv1 | Illumina | 36.22 | MACH v1.0.16 | ProbABEL |
| LBC1936 | Illumina Human 610_Quadv1 | Illumina | 36.22 | MACH v1.0.16 | ProbABEL |
| MARTHA | Illumina Human 610_Quadv1 | Illumina | 35.21 | MACH v1.0.16 | Plink, ProbABEL |
| NTR | Affymetrix / Perlegen 600K; Illumina Human 660, Illumina 370K | Perlegen Proprietary,  Illumina Beadstudio | 36.2 | IMPUTE v0.5 | Quicktest |
| ORCADES | Illumina HumanHap300 v2 Duo | Illumina | 36.22 | MACH v1.0.16 | ProbABEL |
| PROCARDIS-CL | Illumina 1M, Illumina 610 Quad | Beadstudio | 36 | MACH  v1.0.16 | STATA |
| PROCARDIS-Im | Illumina 1M, Illumina 610 Quad | Beadstudio | 36 | MACH  v1.0.16 | STATA |
| PROSPER | Illumina Human 660_Quadv1 | Illumina | 36.22 | MACH  v1.0.16 | ProbABEL |
| RS | V3 Illlumina Infinium II HumanHap550 | Illumina BeadStudio | 36.22 | MACH  v1.0.15 | ProbABEL |
| SardiNIA | Affymetrix 10K+500K+6.0 | BLRMM (10K, 500K),  Birdseed (6.0) | 36.3 | MACH v1.0.10 | ProbABEL |
| SHIP | Affymetrix 6.0 (1000K) | Birdseed2 | 36.1 | IMPUTE v0.5.0 | Quicktest |
| WGHS | Illumina HumanHap 300 Duo Plus | Beadstudio | 36 | MACH  v1.0.16 | ProbaABEL |

** LME model was applied using GWAF which is an R package for genome-wide association analyses with family data (Chen MH and Yang Q. GWAF: an R package for genome-wide association analyses with family data. Bioinformatics 2010; 26: 580-581.*
