# Supplementary material for: No Evidence for Genome-Wide Interactions on Plasma Fibrinogen by Smoking, Alcohol Consumption and Body Mass Index: Results from Meta-Analyses of 80,607 Subjects
Source: PLoS One. 2014 Dec 31;9(12):e111156. doi: 10.1371/journal.pone.0111156 (PMC4281156; doi:10.1371/journal.pone.0111156)
Supplement: S3 Table — Genomic inflation factor per study. (DOC) [file pone.0111156.s005.doc]

**Table S3. Genomic inflation factor per study.**

|  | **Smoking status** | **Alcohol consumption** | **BMI** |
| --- | --- | --- | --- |
| ARIC | 1.112 | 1.024 | 1.118 |
| B58C | 1.003 | 0.998 | 1.024 |
| CARDIA | 1.032 | NA | 1.110 |
| CHS | 1.026 | 1.009 | 1.020 |
| CROATIA-Vis | 0.972 | 1.038 | 1.717 |
| FHS | 1.016 | 1.159 | 1.000 |
| HBCS | 1.025 | 1.010 | 1.015 |
| InCHIANTI | 1.032 | 1.018 | 1.054 |
| KORA F3 | 1.068 | 1.069 | 1.111 |
| KORA F4 | 1.382 | 1.049 | 1.148 |
| LBC1921 | 1.231 | 0.983 | 0.898 |
| LBC1936 | 1.126 | 1.080 | 1.163 |
| MARTHA | 1.044 | NA | 1.091 |
| NTR | 1.098 | 1.018 | 1.096 |
| ORCADES | 1.100 | 1.063 | 1.092 |
| PROCARDIS-CL | 1.010 | 1.001 | 1.039 |
| PROCARDIS-Im | 1.017 | 0.997 | 1.091 |
| PROSPER/PHASE | 1.004 | 1.011 | 1.018 |
| RS | 0.982 | 1.015 | 1.003 |
| SardiNIA | 0.935 | 0.850 | 0.943 |
| SHIP | 1.005 | 1.087 | 1.115 |
| WGHS | 1.027 | 1.014 | 1.001 |

*Genomic inflation factor > 1.15 or < 1/1.15 are marked red*
