# Supplementary material for: No Evidence for Genome-Wide Interactions on Plasma Fibrinogen by Smoking, Alcohol Consumption and Body Mass Index: Results from Meta-Analyses of 80,607 Subjects
Source: PLoS One. 2014 Dec 31;9(12):e111156. doi: 10.1371/journal.pone.0111156 (PMC4281156; doi:10.1371/journal.pone.0111156)
Supplement: S4 Table — Interaction with alcohol consumption for association with fibrinogen concentration (in g/L) among SNPs associated with circulating fibrinogen (Sabater-Leal et al. 2013). (DOC) [file pone.0111156.s006.doc]

**Table S4. Interaction with alcohol consumption for association with fibrinogen concentration (in g/L) among SNPs associated with circulating fibrinogen (Sabater-Leal et al. 2013).**

| **SNP** | **Chr** | **Position** | **A1*** | **A2** | **% A1** | **Beta (SE)** | **P value** | **N studies** | **Direction**** | **I^2^ index** |
| --- | --- | --- | --- | --- | --- | --- | --- | --- | --- | --- |
| rs1938492 | 1 | 65890417 | A | C | 62.2 | 0.009 (0.005) | 0.070 | 20 | +-+++-+-++--++++-+++ | 0.0% |
| rs4129267 | 1 | 152692888 | T | C | 38.7 | 0.002 (0.005) | 0.667 | 20 | +++-++++-++-+--++--+ | 23.3% |
| rs10157379 | 1 | 245672222 | T | C | 62.3 | 0.008 (0.005) | 0.105 | 19 | -+-++---+++++++++?++ | 0.0% |
| rs12712127 | 2 | 102093093 | A | G | 40.8 | 0.007 (0.005) | 0.153 | 20 | ++++---++-++-++---+- | 0.0% |
| rs6734238 | 2 | 113557501 | A | G | 57.7 | 0.009 (0.005) | 0.061 | 20 | +-+-++--++-+------++ | 33.5% |
| rs715 | 2 | 211251300 | T | C | 68.1 | -0.015 (0.006) | 0.020 | 16 | -??---+?+-+++++-+?-- | 0.0% |
| rs1476698 | 2 | 241945122 | A | G | 64.8 | -0.009 (0.005) | 0.069 | 20 | -----+--+--++-+-++-- | 0.0% |
| rs1154988 | 3 | 137407881 | A | T | 77.8 | 0.005 (0.006) | 0.415 | 20 | +++---+-+++-+-+-+--- | 0.0% |
| rs16844401 | 4 | 3419450 | A | G | 7.7 | 0.005 (0.012) | 0.671 | 17 | ++?+?+-++++++++--?-- | 14.2% |
| rs1800789 | 4 | 155702193 | A | G | 21.2 | 0.002 (0.006) | 0.682 | 20 | -++++-++--+---+++--+ | 52.7% |
| rs11242111 | 5 | 131783957 | A | G | 5.8 | -0.0002 (0.015) | 0.990 | 9 | -?-?-?+?-?-?+???-+?? | 0.0% |
| rs2106854 | 5 | 131797073 | T | C | 20.9 | 0.006 (0.006) | 0.326 | 20 | -++-++-+--+++--++-+- | 20.1% |
| rs10226084 | 7 | 17964137 | T | C | 52.9 | -0.001 (0.005) | 0.821 | 20 | --+--+-++++-+---+-+- | 0.0% |
| rs2286503 | 7 | 22823131 | T | C | 36.2 | -0.002 (0.005) | 0.678 | 20 | -+++++-++++++--++++- | 28.5% |
| rs7464572 | 8 | 145093155 | C | G | 59.1 | 0.001 (0.005) | 0.803 | 18 | -?+++-+?+-------++-+ | 0.0% |
| rs7896783 | 10 | 64832159 | A | G | 48.2 | 0.001 (0.005) | 0.786 | 20 | ++---+++++-------+++ | 37.8% |
| rs1019670 | 11 | 59697175 | A | T | 36.1 | 0.011 (0.005) | 0.032 | 20 | ++--++--++++-+----++ | 0.0% |
| rs7968440 | 12 | 49421008 | A | G | 63.2 | 0.005 (0.005) | 0.330 | 20 | ++--+--+----+++--+++ | 0.0% |
| rs434943 | 14 | 68383812 | A | G | 31.5 | 0.009 (0.006) | 0.137 | 19 | +-++++++++++-+-++?+- | 2.5% |
| rs12915708 | 15 | 48835894 | C | G | 30.6 | 0.001 (0.005) | 0.889 | 20 | +++-+++-++------+++- | 0.0% |
| rs7204230 | 16 | 51749832 | T | C | 70.1 | 0.002 (0.006) | 0.680 | 18 | +?-+++-?--+-+-+----+ | 26.9% |
| rs10512597 | 17 | 70211428 | T | C | 17.7 | -0.001 (0.007) | 0.864 | 19 | +-----+--+--+-++-?-+ | 19.0% |
| rs4817986 | 21 | 39387382 | T | G | 27.9 | 0.007 (0.006) | 0.248 | 18 | ++?++++----+---++?++ | 40.7% |
| rs6010044 | 22 | 49448804 | A | C | 80.0 | -0.005 (0.007) | 0.468 | 18 | ++?+--+----++-?----+ | 0.0% |

SNP: single nucleotide polymorphism, Chr: chromosome, SE: standard error.

** Allele 1 is effect allele, ** The order of studies under “direction” refers to the order of studies in Table 1*
