# Supplementary material for: No Evidence for Genome-Wide Interactions on Plasma Fibrinogen by Smoking, Alcohol Consumption and Body Mass Index: Results from Meta-Analyses of 80,607 Subjects
Source: PLoS One. 2014 Dec 31;9(12):e111156. doi: 10.1371/journal.pone.0111156 (PMC4281156; doi:10.1371/journal.pone.0111156)
Supplement: S5 Table — Interaction with BMI for association with fibrinogen concentration (in g/L) among SNPs associated with circulating fibrinogen (Sabater-Leal et al. 2013). (DOC) [file pone.0111156.s007.doc]

**Table S5.** Interaction with BMI for association with fibrinogen concentration (in g/L) among SNPs associated with circulating fibrinogen (Sabater-Leal et al. 2013).

| **SNP** | **Chr** | **Position** | **A1*** | **A2** | **% A1** | **Beta (SE)** | **P value** | **N studies** | **Direction**** | **I^2^ index** |
| --- | --- | --- | --- | --- | --- | --- | --- | --- | --- | --- |
| rs1938492 | 1 | 65890417 | a | c | 62.3 | -0.002 (0.001) | 0.016 | 22 | --+----++++-++++++--+- | 10.9% |
| rs4129267 | 1 | 152692888 | t | c | 39.1 | -0.002 (0.001) | 0.028 | 22 | --++--+---++---+++---- | 17.1% |
| rs10157379 | 1 | 245672222 | t | c | 62.2 | 0.001 (0.001) | 0.138 | 21 | ++-++--++---+-+---+?++ | 11.0% |
| rs12712127 | 2 | 102093093 | a | g | 40.9 | -0.0003 (0.001) | 0.683 | 22 | +---+-----++--+--+-++- | 0.0% |
| rs6734238 | 2 | 113557501 | a | g | 58.1 | -0.001 (0.001) | 0.208 | 22 | +--+-+-+--++++-++--+-- | 17.7% |
| rs715 | 2 | 211251300 | t | c | 68.4 | 0.0004 (0.001) | 0.673 | 17 | +?+?---+?+--?+--++-?-+ | 0.0% |
| rs1476698 | 2 | 241945122 | a | g | 64.6 | 0.001 (0.001) | 0.107 | 22 | +-+--+-++++--++-+++--+ | 0.0% |
| rs1154988 | 3 | 137407881 | a | t | 77.7 | 0.001 (0.001) | 0.148 | 22 | -++--++-++--++-+-++++- | 0.0% |
| rs16844401 | 4 | 3419450 | a | g | 7.7 | 0.002 (0.002) | 0.294 | 18 | -+??-?+--++-+-++--+?++ | 0.0% |
| rs1800789 | 4 | 155702193 | a | g | 21.1 | 0.001 (0.001) | 0.217 | 22 | --+--++--+-+++--++++++ | 10.3% |
| rs11242111 | 5 | 131783957 | a | g | 5.7 | -0.0001 (0.003) | 0.958 | 9 | +??-?+?-?-?++?-????-?? | 0.0% |
| rs2106854 | 5 | 131797073 | t | c | 20.8 | 0.001 (0.001) | 0.515 | 22 | +-++++--++++-+---+--+- | 31.1% |
| rs10226084 | 7 | 17964137 | t | c | 52.5 | 0.0002 (0.001) | 0.763 | 22 | +-------+----+---+--++ | 11.2% |
| rs2286503 | 7 | 22823131 | t | c | 35.9 | -0.0004 (0.001) | 0.618 | 22 | -++-+-++--++++-+-+-+-- | 29.3% |
| rs7464572 | 8 | 145093155 | c | g | 59.5 | 0.0002 (0.001) | 0.831 | 19 | +?---++-?+-+?--+++++-- | 25.0% |
| rs7896783 | 10 | 64832159 | a | g | 48.2 | -0.0001 (0.001) | 0.886 | 22 | +----+-+--++-+-----++- | 35.0% |
| rs1019670 | 11 | 59697175 | a | t | 36.2 | -0.002 (0.001) | 0.0062 | 22 | --+---++-++++-+-+-++-- | 0.0% |
| rs7968440 | 12 | 49421008 | a | g | 63.6 | -0.001 (0.001) | 0.180 | 22 | --+-++++-++-++++-----+ | 0.0% |
| rs434943 | 14 | 68383812 | a | g | 31.5 | -0.0004 (0.001) | 0.656 | 21 | -+--+-++-+--++-+--+?+- | 26.5% |
| rs12915708 | 15 | 48835894 | c | g | 30.6 | 0.001 (0.001) | 0.259 | 22 | --++-++---++-+--++++++ | 0.0% |
| rs7204230 | 16 | 51749832 | t | c | 70.1 | 0.001 (0.001) | 0.520 | 19 | +?-+-+--?+++?+---+-++- | 0.0% |
| rs10512597 | 17 | 70211428 | t | c | 17.6 | -0.003 (0.001) | 0.0049 | 21 | --+-+-+++--+---+++-?+- | 0.0% |
| rs4817986 | 21 | 39387382 | t | g | 27.9 | -0.002 (0.001) | 0.088 | 20 | +-?--++++-+---+----?-- | 0.0% |
| rs6010044 | 22 | 49448804 | a | c | 79.9 | 0.001 (0.001) | 0.567 | 21 | ++++-+--++--+-++?++-+- | 0.0% |
