# Supplementary material for: No Evidence for Genome-Wide Interactions on Plasma Fibrinogen by Smoking, Alcohol Consumption and Body Mass Index: Results from Meta-Analyses of 80,607 Subjects
Source: PLoS One. 2014 Dec 31;9(12):e111156. doi: 10.1371/journal.pone.0111156 (PMC4281156; doi:10.1371/journal.pone.0111156)
Supplement: S1 Funding — Funding statement. (DOC) [file pone.0111156.s008.doc]

**Funding**

The Atherosclerosis Risk in Communities Study (**ARIC**) is carried out as a collaborative study supported by National Heart, Lung, and Blood Institute contracts

(HHSN268201100005C, HHSN268201100006C, HHSN268201100007C, HHSN268201100008C, HHSN268201100009C, HHSN268201100010C, HHSN268201100011C, and HHSN268201100012C), R01HL087641, R01HL59367 and R01HL086694; National Human Genome Research Institute contract U01HG004402; and National Institutes of Health contract HHSN268200625226C. The authors thank the staff and participants of the ARIC study for their important contributions. We would like to thank the University of Minnesota Supercomputing Institute for use of the calhoun supercomputers. Infrastructure was partly supported by Grant Number UL1RR025005, a component of the National Institutes of Health and NIH Roadmap for Medical Research. Additional funding for genotyping came from NIH grant R01 HL59367.

We acknowledge use of phenotype and genotype data from the British 1958 Birth Cohort (**B58C**) DNA collection, funded by the Medical Research Council grant G0000934 and the Wellcome Trust grant 068545/Z/02 (http://www.b58cgene.sgul.ac.uk/). Genotyping for the B58C-WTCCC subset was funded by the Wellcome Trust grant 076113/B/04/Z. The B58C-T1DGC genotyping utilized resources provided by the Type 1 Diabetes Genetics Consortium, a collaborative clinical study sponsored by the National Institute of Diabetes and Digestive and Kidney Diseases (NIDDK), National Institute of Allergy and Infectious Diseases (NIAID), National Human Genome Research Institute (NHGRI), National Institute of Child Health and Human Development (NICHD), and Juvenile Diabetes Research Foundation International (JDRF) and supported by U01 DK062418. B58C-T1DGC GWAS data were deposited by the Diabetes and Inflammation Laboratory, Cambridge Institute for Medical Research (CIMR), University of Cambridge, which is funded by Juvenile Diabetes Research Foundation International, the Wellcome Trust and the National Institute for Health Research Cambridge Biomedical Research Centre; the CIMR is in receipt of a Wellcome Trust Strategic Award (079895). The B58C-GABRIEL genotyping was supported by a contract from the European Commission Framework Programme 6 (018996) and grants from the French Ministry of Research.

The Coronary Artery Risk Development in Young Adults (**CARDIA)** study is funded by contracts N01-HC-95095, N01-HC-48047, N01-HC-48048, N01-HC-48049, N01-HC-48050, N01-HC-45134, N01-HC-05187, N01-HC-45205, and N01-HC-45204 from the National Heart, Lung, and Blood Institute to the CARDIA investigators. Genotyping of the CARDIA participants was supported by grants U01-HG-004729, U01-HG-004446, and U01-HG-004424 from the National Human Genome Research Institute. Statistical analyses were supported by grants U01-HG-004729 and R01-HL-084099 to MF.

The Cardiovascular Health Study (**CHS**) was supported by NHLBI contracts HHSN268201200036C, HHSN268200800007C, N01 HC55222, N01HC85079, N01HC85080, N01HC85081, N01HC85082, N01HC85083, N01HC85086, and grant HL080295, with additional contribution from the National Institute of Neurological Disorders and Stroke (NINDS). Additional support was provided through AG023629 from the National Institute on Aging (NIA). A full list of CHS investigators and institutions can be found at http://www.chs-nhlbi.org/pi.htm [www.chs-nhlbi.org]. DNA handling and genotyping at Cedars-Sinai Medical Center was supported in part by the National Center for Research Resources, grant UL1RR033176, and is now at the National Center for Advancing Translational Sciences, CTSI grant UL1TR000124; in addition to the National Institute of Diabetes and Digestive and Kidney Disease grant DK063491 to the Southern California Diabetes Endocrinology Research Center.

The **CROATIA-Vis** study in the Croatian island of Vis was supported through the grants from the Medical Research Council UK and Ministry of Science, Education and Sport of the Republic of Croatia (number 108-1080315-0302) and the European Union framework program 6 EUROSPAN project (contract no. LSHG-CT-2006-018947).

The Framingham Heart Study (**FHS**) was supported by the National Heart, Lung and Blood Institute's Framingham Heart Study (Contract No. N01-HC-25195) and its contract with Affymetrix, Inc for genotyping services (Contract No. N02-HL-6-4278).

The Helsinki Birth Cohort Study (**HBCS**) has been supported by grants from the Academy of Finland, the Finnish Diabetes Research Society, Folkhälsan Research Foundation, Novo Nordisk Foundation, Finska Läkaresällskapet, Signe and Ane Gyllenberg Foundation, University of Helsinki, European Science Foundation (EUROSTRESS), Ministry of Education, Ahokas Foundation, Emil Aaltonen Foundation, Juho Vainio Foundation, and Wellcome Trust (grant number WT089062). We thank all study participants as well as everybody involved in the Helsinki Birth Cohort Study.

The **InCHIANTI** study baseline (1998-2000) was supported as a "targeted project" (ICS110.1/RF97.71) by the Italian Ministry of Health and in part by the U.S. National Institute on Aging (Contracts: 263 MD 9164 and 263 MD 821336).

The **KORA** research platform (KORA, Cooperative Research in the Region of Augsburg) was initiated and financed by the Helmholtz Zentrum München -German Research Center for Environmental Health, Neuherberg, Germany, which is funded by the German Federal Ministry of Education and Research and by the State of Bavaria. Part of this work was financed by the German National Genome Research Network (NGFNplus, project number 01GS0834) and through additional funds from the University of Ulm. Furthermore, KORA research was supported within the Munich Center of Health Sciences (MC Health), Ludwig-Maximilians-Universität München, as part of LMUinnovativ.

The Lothian Birth Cohorts 1921 and 1936 (**LBC1921 and LBC1936**) whole genome association study was funded by the Biotechnology and Biological Sciences Research Council (BBSRC). The LBC1936 research was supported by a programme grant from Research Into Ageing and continues with programme grants from Help the Aged/Research Into Ageing (Disconnected Mind). The LBC1921 data collection was funded by the BBSRC. The study was conducted within the University of Edinburgh Centre for Cognitive Ageing and Cognitive Epidemiology, supported by the BBSRC, Engineering and Physical Sciences Research Council (EPSRC), Economic and Social Research Council (ESRC), and Medical Research Council (MRC), as part of the cross-council Lifelong Health and Wellbeing Initiative (G0700704/84698).

The **MARTHA** project was supported by a grant from the Program Hospitalier de Recherche Clinique. Tiphaine Oudot-Mellakh was supported by a grant from the Fondation pour la Recherche Médicale. Statistical analyses in MARTHA benefit from the C2BIG computing centre funded by the Fondation pour la Recherche Médicale, La Région Ile de France (CODDIM) and the Genomic Network of the Pierre and Marie Curie University (Paris 06)."

For the Netherlands Twin Registry (**NTR**), funding was obtained from the Netherlands Organization for Scientific Research (NWO: MagW/ZonMW grants 904-61-090, 985-10-002, 904-61-193,480-04-004, 400-05-717, Addiction-31160008, Middelgroot-911-09-032, Spinozapremie 56-464-14192), Center for Medical Systems Biology (CSMB, NWO Genomics), NBIC/BioAssist/RK(2008.024), Biobanking and Biomolecular Resources Research Infrastructure (BBMRI –NL, 184.021.007), the VU University’s Institute for Health and Care Research (EMGO+ ) and Neuroscience Campus Amsterdam (NCA), the European Science Foundation (ESF, EU/QLRT-2001-01254), the European Community's Seventh Framework Program (FP7/2007-2013), ENGAGE (HEALTH-F4-2007-201413); the European Science Council (ERC Advanced, 230374), Rutgers University Cell and DNA Repository (NIMH U24 MH068457-06), the Avera Institute, Sioux Falls, South Dakota (USA), and the National Institutes of Health (NIH, R01D0042157-01A, Grand Opportunity grants 1RC2MH089951-01 and 1RC2 MH089995-01). Part of the genotyping and analyses were funded by the Genetic Association Information Network (GAIN) of the Foundation for the National Institutes of Health.

**ORCADES** was supported by the Chief Scientist Office of the Scottish Government, the Royal Society and the European Union framework program 6 EUROSPAN project (contract no. LSHG-CT-2006-018947). DNA extractions were performed at the Wellcome Trust Clinical Research Facility in Edinburgh.

**PROCARDIS** was supported by the European Community Sixth Framework Program (LSHM-CT- 2007-037273), AstraZeneca, the British Heart Foundation, the Wellcome Trust (Contract No. 075491/Z/04), the Swedish Research Council, the Knut and Alice Wallenberg Foundation, the Swedish Heart-Lung Foundation, the Torsten and Ragnar Söderberg Foundation, the Strategic Cardiovascular Program of Karolinska Institutet and Stockholm County Council, the Foundation for Strategic Research and the Stockholm County Council. Maria Sabater-Lleal is a recipient of a Marie Curie Intra European Fellowship within the 7th Framework Programme of the European Union (PIEF-GA-2009-252361). Bengt Sennblad acknowledges funding from Magnus Bergvall's foundation and the Foundation for old servants.

The **PROSPER**/PHASE study was supported by an investigator initiated grant obtained from Bristol-Myers Squibb. Prof. Dr. J. W. Jukema is an Established Clinical Investigator of the Netherlands Heart Foundation (grant 2001 D 032). Support for genotyping was provided by the seventh framework program of the European commission (grant 223004) and by the Netherlands Genomics Initiative (Netherlands Consortium for Healthy Aging grant 050-060-810). Fibrinogen assays were funded through a grant from the Scottish Executive Chief Scientist Office, Health Services Research Committee grant number CZG/4/306.

The Rotterdam Study (**RS**) is supported by the Erasmus Medical Center and Erasmus University Rotterdam; the Netherlands Organization for Scientific Research; the Netherlands Organization for Health Research and Development (ZonMw); the Research Institute for Diseases in the Elderly; The Netherlands Heart Foundation; the Ministry of Education, Culture and Science; the Ministry of Health Welfare and Sports; the European Commission; and the Municipality of Rotterdam. Support for genotyping was provided by the Netherlands Organization for Scientific Research (NWO) (175.010.2005.011, 911.03.012) and Research Institute for Diseases in the Elderly (RIDE). This study was further supported by the Netherlands Genomics Initiative (NGI)/Netherlands Organisation for Scientific Research (NWO) project nr. 050-060-810. Dr. Dehghan is supported by NWO grant (veni, 916.12.154) and the EUR Fellowship. Dr. Kavousi is supported by AXA Research Fund. Oscar H. Franco works in ErasmusAGE, a center for aging research across the life course funded by Nestle Nutrition (Nestec Ltd), Metagenics Inc, and AXA.

The **SardiNIA** team was supported by Contract NO1-AG-1–2109 from the NIA. We thank the many individuals who generously participated in this study, the Mayors and citizens of the Sardinian towns involved, the head of the Public Health Unit ASL4, and the province of Ogliastra for their volunteerism and cooperation. In addition, we are grateful to the Mayor and the administration in Lanusei for providing and furnishing the clinic site. We are grateful to the physicians Marco Orrù, Maria Grazia Pilia, Liana Ferreli, Francesco Loi, nurses Paola Loi, Monica Lai and Anna Cau who carried out participant physical exams; the recruitment personnel Susanna Murino; Mariano Dei, Sandra Lai, Antonella Mulas, Gianluca Usala, Andrea Maschio, Fabio Busonero for genotyping; Maria Grazia Piras and Monica Lobina for fibrinogen phenotyping. Finally, we thank the HPC Group at the CRS4 center in Pula, Sardinia, for access to the computing cluster facility.

The **SHIP** study is part of the Community Medicine Research net of the University of Greifswald, Germany, which is funded by the Federal Ministry of Education and Research (grants no. 01ZZ9603, 01ZZ0103, and 01ZZ0403), the Ministry of Cultural Affairs as well as the Social Ministry of the Federal State of Mecklenburg - West Pomerania. Genome-wide data have been supported by the Federal Ministry of Education and Research (grant no. 03ZIK012) and a joint grant from Siemens Healthcare, Erlangen, Germany and the Federal State of Mecklenburg West Pomerania. The University of Greifswald is a member of the 'Center of Knowledge Interchange' program of the Siemens AG and the Caché Campus program of the InterSystems GmbH. This work is also part of the research project Greifswald Approach to Individualized Medicine (GANI_MED). The GANI_MED consortium is funded by the Federal Ministry of Education and Research and the Ministry of Cultural Affairs of the Federal State of Mecklenburg – West Pomerania (03IS2061A). Computing resources have been made available by the Leibniz Supercomputing Centre of the Bavarian Academy of Sciences and Humanities (HLRB project h1231).

The Women's Genome Health Study (**WGHS)** is supported by HL 043851 and HL080467 from the National Heart, Lung, and Blood Institute and CA 047988 from the National Cancer Institute, the Donald W. Reynolds Foundation and the Foundation Leducq, with collaborative scientific support and funding for genotyping provided by Amgen.

The funders had no role in study design, data collection and analysis, decision to publish, or preparation of the manuscript.
