# Supplementary material for: No Evidence for Genome-Wide Interactions on Plasma Fibrinogen by Smoking, Alcohol Consumption and Body Mass Index: Results from Meta-Analyses of 80,607 Subjects
Source: PLoS One. 2014 Dec 31;9(12):e111156. doi: 10.1371/journal.pone.0111156 (PMC4281156; doi:10.1371/journal.pone.0111156)
Supplement: S1 Methods — This file gives additional information about the study samples, fibrinogen measurements, genotyping/imputation and statistical analyses. (DOC) [file pone.0111156.s010.doc]

**Methods S1**

**Study samples**

The **Atherosclerosis Risk in Communities (ARIC)** study is a longitudinal cohort study of atherosclerosis and its clinical sequelae. It recruited a population-based sample of 15,792 men and women aged 45-64 years from four US communities in 1987-89 [1]. The present analyses were restricted to subjects of European decent.

The **British 1958 birth cohort (B58C**) is a national population sample followed periodically from birth. At age 44-45 years, 9377 cohort members were examined by a research nurse in the home as described previously [2] and non-fasting blood samples were collected with permission for DNA extraction and creation of immortalised cell cultures (http://www.b58cgene.sgul.ac.uk/collection.php). DNA samples from unrelated subjects of white ethnicity, with nationwide geographic coverage, were genotyped either by the Wellcome Trust Case Control Consortium (WTCCC) [3], the Type 1 Diabetes Genetics Consortium [4] or the GABRIEL consortium [5].

The **Coronary Artery Risk Development in Young Adults (CARDIA)** Study is a prospective multicenter study with 5,115 adults Caucasian and African American participants of the age group 18-30 years, recruited from four centers. The recruitment was done from the total community in Birmingham, AL, from selected census tracts in Chicago, IL and Minneapolis, MN; and from the Kaiser Permanente health plan membership in Oakland, CA. The details of the study design for the CARDIA study have been published before [6]. Eight examinations have been completed since initiation of the study in 1985–1986, respectively in the years 0, 2, 5, 7, 10, 15, 20, and 25. Written informed consent was obtained from participants at each examination and all study protocols were approved by the institutional review boards of the participating institutions.

The **Cardiovascular Health Study (CHS)** is a population-based cohort study of risk factors for CHD and stroke in adults ≥65 years conducted across 4 field centers [7]. The original predominantly Caucasian cohort of 5,201 persons was recruited in 1989-1990 from random samples of the Medicare eligibility lists; subsequently, an additional predominantly African-American cohort of 687 persons were enrolled subsequently for a total sample of 5,888. DNA was extracted from blood samples drawn on all participants at their baseline examination in 1989-90. All subjects provided informed consent to use their DNA samples for cardiovascular genetic research.

The **CROATIA-Vis** study, Croatia, is a family-based, cross-sectional study in the isolated island of Vis [8] that included 1,056 examinees aged 18-93. Blood samples were collected in 2003 and 2004 along with many clinical and biochemical measures and lifestyle and health questionnaires.

The **Framingham Heart Study (FHS)** started in 1948 with 5,209 randomly ascertained participants from Framingham, Massachusetts, US, who had undergone biannual examinations to investigate cardiovascular disease and its risk factors. In 1971, the Offspring cohort (comprising 5,124 children of the original cohort and the children's spouses) and in 2002, the Third Generation (consisting of 4,095 children of the Offspring cohort) were recruited. FHS participants in this study are of European ancestry. The methods of recruitment and data collection for the Offspring and Third Generation cohorts have been described [9].

The **Helsinki Birth Cohort Study (HBCS)** is composed of 8,760 individuals born between the years 1934-44 in one of the two main maternity hospitals in Helsinki, Finland. Between 2001 and 2003, a randomly selected sample of 928 males and 1 075 females participated in a clinical follow-up study with a focus on cardiovascular, metabolic and reproductive health, cognitive function and depressive symptoms. Weight and height were measured during a clinical visit and blood was drawn. Smoking and alcohol were self-reported. Detailed information on the selection of the HBCS participants and on the study design can be found elsewhere [10-12]. Research plan of the HBCS was approved by the Institutional Review Board of the National Public Health Institute and all participants have signed an informed consent. After excluding subjects with anticoagulant medication (n=308) 1,400 subjects with valid phenotype and genotype data (40.2% men) remained. The mean age of the subjects was 61.4 (SD=2.9).

The **InCHIANTI study** is a population-based epidemiological study aimed at evaluating the factors that influence mobility in the older population living in the Chianti region in Tuscany, Italy. The details of the study have been previously reported [13]. Briefly, 1,616 residents were selected from the population registry of Greve in Chianti (a rural area: 11,709 residents with 19.3% of the population greater than 65 years of age), and Bagno a Ripoli (Antella village near Florence; 4,704 inhabitants, with 20.3% greater than 65 years of age). The participation rate was 90% (n=1,453), and the subjects ranged between 21-102 years of age. Overnight fasted blood samples were for genomic DNA extraction, and measurement of fibrinogen. Illumina Infinium HumanHap 550K SNP arrays were used for genotyping [14]. Genome-wide association of genotyped and imputed SNPs was conducted using MERLIN as described previously [15].

The **KORA Augsburg Study** **(KORA F3 and KORA F4)** consisted of a series of independent population-based epidemiological surveys of participants living in the region of Augsburg, Southern Germany [16]. All survey participants are residents of German nationality identified through the registration office. The presented data were derived from the third and fourth population-based Monitoring of Trends and Determinants in Cardiovascular Disease (MONICA)/Cooperative Health Research in the Region of Augsburg (KORA) surveys S3 and S4. These cross-sectional surveys covering the city of Augsburg (Germany) and two adjacent counties were conducted in 1994/95 (S3) and 1999/2001 (S4) with 4,856 (S3) and 4,261 (S4) individuals aged 25 to 74 years. S3 was part of the WHO MONICA study. In a follow-up examination of S3 conducted in 2004/05 (KORA F3) and of S4 conducted in 2006/08 (KORA F4), a number of 3,006 (F3) and 3,080 (F4) subjects participated. All participants underwent standardized examinations including blood withdrawals for plasma and DNA. For the MONICA/KORA genome-wide association study, a number of 1,644 and 1,814 subjects were selected from F3 and F4 samples [17]. After excluding subjects with no information on fibrinogen, smoking status, alcohol consumption and BMI, the final populations for the MONICA/KORA data comprised 1,520 (S3/F3) and 1,777 (S4/F4) subjects.

The **LBC1921** cohort consists of 550 relatively healthy individuals, 316 females and 234 males, assessed on cognitive and medical traits at 79 years of age. They were born in 1921, most took part in the Scottish Mental Survey of 1932, and almost all lived independently in the Lothian region (Edinburgh City and surrounding area) in Scotland. When tested, the sample had a mean age of 79.1 years (SD = 0.6). A full description of participant recruitment and testing can be found elsewhere [18]. The **LBC1936** consists of 1,091 relatively healthy individuals assessed on cognitive and medical traits at 70 years of age. They were born in 1936, most took part in the Scottish Mental Survey of 1947, and almost all lived independently in the Lothian region of Scotland. The sample of 548 men and 543 women had a mean age 69.6 years (SD = 0.8). A full description of participant recruitment and testing can be found elsewhere [19]. Ethics permission for the study was obtained from the Multi-Centre Research Ethics Committee for Scotland (MREC/01/0/56) and from Lothian Research Ethics Committee (LBC1936: LREC/2003/2/29 and LBC1921: LREC/1998/4/183). The research was carried out in compliance with the Helsinki Declaration. All subjects gave written, informed consent.

After excluding individuals on anti-coagulation therapy and without information on fibrinogen, smoking status, alcohol consumption and BMI, the final sample for LBC1921 was 466 (196 males) and LBC1936 was 989 (502 males).

The **MARseille THrombosis Association (MARTHA)** project has been previously described [20,21] Briefly, MARTHA consist in two independent samples of VT patients, named MARTHA08 (N=1,006) and MARTHA10 (N=586). MARTHA patients are unrelated subjects of European origin, with the majority being of French ancestry, consecutively recruited at the Thrombophilia center of La Timone hospital (Marseille, France) between January 1994 and October 2005. All patients had a documented history of VT and free of well characterized genetic risk factors including AT, PC, or PS deficiency, homozygosity for FV Leiden or FII 20210A, and lupus anticoagulant. They were interviewed by a physician on their medical history, which emphasized manifestations of deep vein thrombosis and pulmonary embolism using a standardized questionnaire. The thrombotic events were confirmed by venography, Doppler ultrasound, spiral computed tomographic scanning angiography, and/or ventilation/perfusion lung scan.

Between January 2004 and July 2008, 9,530 participants from 3,477 families registered in the **Netherlands Twin Registry (NTR)** were visited at home between 7:00 and 10:00 am for collection of blood samples. Fertile women were bled on day 2–4 of the menstrual cycle, or in their pill-free week. Body composition was measured and information about physical health and lifestyle (e.g. smoking and drinking behavior, physical exercise, medication use) was obtained. For more detailed information about the methodology of the NTR Biobank study, see Willemsen et al [22]. Valid GWA data were available for 2,490 individuals.

The **Orkney Complex Disease Study (ORCADES)** was performed in the Scottish archipelago of Orkney[23] and collected data between 2005 and 2011 Data for 889 participants aged 18 to 100 years from a subgroup of ten islands, were used for this analysis.

The **Precocious Coronary Artery Disease Study (PROCARDIS)** is a multicentric European project, which assembled uniquely large and carefully phenotyped collections of subjects with coronary artery disease (CAD) cases and controls from four European countries (UK, Italy, Sweden and Germany). CAD (defined as myocardial infarction, acute coronary syndrome, unstable or stable angina, or need for coronary artery bypass surgery or percutaneous coronary intervention) was diagnosed before 66 years of age and 80% of cases had a sibling fulfilling the same criteria for CAD. Subjects with self-reported non-European ancestry were excluded. Among the “genetically-enriched” CAD cases, 70% had suffered myocardial infarction (MI) [24].

A detailed description of the **PROspective Study of Pravastatin in the Elderly at Risk (PROSPER)** study has been published elsewhere [25,26] PROSPER was a prospective multicenter randomized placebo-controlled trial to assess whether treatment with pravastatin diminishes the risk of major vascular events in elderly. Between December 1997 and May 1999, we screened and enrolled subjects in Scotland (Glasgow), Ireland (Cork), and the Netherlands (Leiden). Men and women aged 70-82 years were recruited if they had pre-existing vascular disease or increased risk of such disease because of smoking, hypertension, or diabetes. A total number of 5,804 subjects were randomly assigned to pravastatin or placebo. A large number of prospective tests were performed including Biobank tests and cognitive function measurements.

The **Rotterdam Study (RS)** is a prospective, population-based cohort study to investigate the determinants of chronic diseases among participants aged 55 years and older living in the well-defined district of Ommord in the municipality of Rotterdam, the Netherlands [27]. Subjects are of European ancestry based on their self-report. The baseline examination was completed in 1990-1993 and consisted of 7,983 participants. The current study was based on 2,068 individuals with available fibrinogen measurements and genotype data. The Medical Ethics Committee of Erasmus MC approved the study, and all participants gave informed consent.

The **SardiNIA study** is a large population-based study which consists of 6,148 individuals, males and females, ages 14-102 y, that were recruited from a cluster of four towns in the Lanusei Valley of Sardinia [28]. Samples have been characterized for several quantitative traits and medical conditions, including fibrinogen. The final dataset for genome-wide analyses consisted of 4,691 individuals phenotyped for fibrinogen levels.

The **Study of Health in Pomerania (SHIP)** is a longitudinal cohort study in West Pomerania in the north-east area of Germany [29]. From the entire study population of 212,157 inhabitants living in the area, a sample was selected from the population registration offices, where all German inhabitants are registered. Only individuals with German citizenship and main residency in the study area were included. A two-stage cluster sampling method was adopted from the WHO MONICA Project Augsburg, Germany. In a first step, the three cities of the region (with 17,076 to 65,977 inhabitants) and the 12 towns (with 1,516 to 3,044 inhabitants) were selected. Further 17 out of 97 smaller towns (with less than 1,500 inhabitants) were drawn at random. In a second step, from each of the selected communities, subjects were drawn at random, proportional to the population size of each community and stratified by age and gender. Finally, 7,008 subjects aged 20 to 79 years were sampled, with 292 persons of each gender in each of the twelve five-year age strata. In order to minimize drop-outs by migration or death, subjects were selected in two waves. The net sample (without migrated or deceased persons) comprised 6,267 eligible subjects. Selected persons received a maximum of three written invitations. In case of non-response, letters were followed by a phone call or by home visits if contact by phone was not possible. The SHIP population finally comprised 4,308 participants at baseline (corresponding to a final response of 68.8%).

The **Women’s Genome Health Study (WGHS)** is a prospective population-based cohort of North American women who consented for genetic analysis [30]. The WGHS is derived from the Women’s Health Study, a randomized placebo-controlled trial of aspirin for primary prevention of cardiovascular disease among healthy women who were at least 45 years of age at enrollment (1992-95) and were followed for 10 years for incident cardiovascular disease. Blood samples were provided at baseline and extensive demographic and clinical data have been collected since enrollment. The final analytic samples consist of women with confirmed, self-reported European ancestry, genome-wide genotyping data, and non-missing fibrinogen and covariate measurements. The final sample sizes for each analysis is as follows: GxSmoke: n=23,062; GxAlcohol: n=23,076; GxBMI: n=22,677.

**Fibrinogen measurement methods**

**ARIC:** Fibrinogen was measured at baseline in the entire ARIC cohort after an 8-hour fasting period. Circulating plasma fibrinogen was measured by the Clauss clotting rate method [31]. A total of 11,362 European American participants had data available for fibrinogen after warfarin users were excluded. Participants whose fibrinogen measurement was off 6SD from the mean were also excluded.

**B58C:** Details of the blood collection, fibrinogen measurement and covariate adjustment have been described elsewhere [32]. In brief, fibrinogen was measured by the Clauss method using an MDA 180 coagulometer (Biomerieux, Basingstoke, UK).

**CARDIA:** Fibrinogen was measured using an immunoassay on plasma samples from CARDIA participants at the year 7 examination. Total fibrinogen concentration for this assay was determined at the University of Vermont using immunonephelometry (BNII Nephelometer 100 Analyzer; Dade Behring, Deerfield, IL, USA). The amount of immuno-reactive fibrinogen present in the sample was quantitatively determined by light scatter intensity, and the results reported in mg/dL. The intra-assay and inter-assay coefficient of variation (CVs) for the immunoassay were 2.7% and 2.6%, respectively.

**CHS:** After an 8-12-h fast, CHS participants underwent phlebotomy by atraumatic venipuncture with a 21-gauge butterfly needle connected to a Vacutainer (Becton Dickinson, Rutherford, NJ) outlet via a Luer adaptor [33]. For fibrinogen determination, an additional citrate-containing tube was processed at 4° C. The study measured fibrinogen levels using the Clauss method.

**CROATIA-Vis:** Clauss method for measuring plasma fibrinogen.

**FHS:** Fibrinogen levels were measured using the Clauss method in the offspring and the third-generation subjects, and a modified method of Ratnoff and Menzie in the original cohort subjects [34].

**HBCS:** Fibrinogen levels were measured in g/L using the Clauss method with an electrical impedance end point.

**InCHIANTI:** Plasma fibrinogen concentrations were measured by the Clauss method using STA fibrinogen assay (Diagnostic Stago, Roche Diagnostics, France). The intra- and inter- assay CV was 4.1%.

**KORA F3 and KORA F4:** Fibrinogen was determined by an immunonephelometric method (Dade Behring Marburg GmbH, Marburg, Germany) on a Behring Nephelometer II analyzer.

**LBC1921 and LBC1936:** Fibrinogen levels were measured using HemosIL^TM^ based on the Clauss method. No transformations were applied. Outliers were removed (>3.3SD).

**MARTHA:** Blood samples were collected by antecubital venipuncture into Vacutainer® tubes 0.105 M trisodium citrate (ratio 9:1, Becton Dickinson) for the coagulation test and the thrombin generation assay. Platelet-poor plasma (PPP) was obtained after double centrifugation of citrated blood (3000 g for 10 min at 25°C) and kept frozen at -80°C until analysis. Fibrinogen levels were measured using the Clauss method on STAR automatic coagulomater. After excluding individuals under anti-coagulant therapy, fibrinogen levels were available in 613 patients.

**NTR:** Fibrinogen was measured in a 4.5 ml CTAD tube that was stored during transport in melting ice and upon arrival at the laboratory, centrifuged for 20 minutes at 2000x g at 4° C, after which citrated plasma was harvested, aliquoted (0.5 ml), snapfrozen in dry ice, and stored at –30° C. Fibrinogen levels were determined on a STA Compact Analyzer Diagnostica Stago, France), using STA Fibrinogen (Diagnostica Stago, France).

**ORCADES:** Clauss method for measuring plasma fibrinogen.

**PROCARDIS:** For a subset of 2,788 individuals from the PROCARDIS sample, plasma fibrinogen concentrations were measured in fasting citrate plasma samples by the Clauss method using the IL Test Fibrinogen C kit and IL Test Calibration Plasma, on the ACL-9000 coagulometer (all from Instrumentation Laboratory Spa, Milan, Italy). The inter-assay CV was 7% (n=106). This sample is denoted as PROCARDIS-CL.

For a second non-overlapping subset of PROCARDIS individuals (n=3,387), fibrinogen was measured in EDTA plasma samples using Dade Behring reagents on the Dade-Behring Nephelometer II analyzer (Dade-Behring, Marburg, Germany). The inter-assay CV was 5.5%. This sample is denoted as PROCARDIS-Im.

**PROSPER:** Fibrinogen levels were measured by the Clauss method (MDA180 coagulometer; Trinity Biotech; calibrant 9th British standard National Institute for Biological Standards and Control).

**RS:** Fibrinogen levels were derived from the clotting curve of the prothrombin time assay using Thromborel S as a reagent on an automated coagulation laboratory 300 (ACL 300, Instrumentation Laboratory, Zaventem, Belgium).

**SardiNIA:** Fibrinogen levels were measured using the Clauss method.

**SHIP:** For the laboratory examinations, non-fasting blood samples were drawn from the cubital vein in the supine position between 07:00 a.m. and 04:00 p.m. Plasma fibrinogen concentrations were assayed according to Clauss using an Electra 1600 analyzer (Instrumentation Laboratory, Barcelona, Spain) calibrated and maintained according to the manufacturers’ instructions. Quality control was performed internally as well as externally by participating in external proficiency testing programs.

**WGHS:** Fibrinogen was measured by a mass-based immunoturbidimetric assay (DiaSorin) with reproducibility of 5.20% and 3.99% at concentrations of 0.99 and 2.74 g/L respectively.

**Genotyping methods**

**ARIC:** Genotyping was performed using the Affymetrix Genome-Wide Human SNP Array 6.0 (Affymetrix, Santa Clara, CA, USA) at the Broad Institute of Harvard and MIT. Exclusions at individual level included disallowing DNA use, unintentional duplicates with higher missing genotype rates, suspected mixed/contaminated samples, scans from one problem plate, samples with a mismatch between called and phenotypic sex, samples with genotype mismatch with 39 previously genotyped SNPs, suspected first‐degree relative of an included individual, and genetic outliers based on average IBS statistics and principal components analysis using EIGENSTRAT [35]. SNPs were excluded due to no chromosome location, being monomorphic, call rate < 95%, or HWE-p < 10^-6^ for SNPs with MAF>0.05. In addition, imputation to approximately 2.5 million autosomal SNPs identified in HapMap Phase II CEU samples was performed using MACH v1.0.16. SNPs that met the following criteria were included in the imputation: MAF ≥ 1%, call rate ≥ 95%, and HWE-p value ≥ 10^-5^.

**B58C:** Genotyping was performed using the Illumina HumanHap chip arrays: either 550K (WTCCC [3] and T1DGC [4]) or 610K (GABRIEL [5]). Pre-imputation inclusion filters were: SNPs common to all three deposits, with similar allele frequencies (p>0.0001 on pairwise comparisons), >95% call rate, >1% minor allele frequency and p>0.0001 on testing for Hardy-Weinberg equilibrium. 482,570 autosomal SNPs were included as inputs to the imputation step. Genotypes at other loci were imputed by MACH version 1.0.16 [36,37] using the HapMap CEU r21 reference haplotypes.

**CARDIA:** CARDIA Study samples were genotyped using the Affymetrix Genome-Wide Human SNP Array 6.0 (Santa Clara, California); only participants of European descent were included in the GWAS analyses. Genotyping was completed for 1,720 individuals with a sample call rate ≥ 98%. A total of 578,568 SNPs passed quality control (MAF ≥ 2%, call rate ≥ 95%, HWE ≥ 10^-4^) and were used for imputation.

**CHS:** In 2007-2008, genotyping was performed on CHS European-ancestry participants at the General Clinical Research Center's Phenotyping/Genotyping Laboratory at Cedars-Sinai using the Illumina 370CNV BeadChip system on 3,980 CHS participants who were free of CVD at baseline, consented to genetic testing, and had DNA available for genotyping. In 2010, the African-ancestry were genotyped at the same lab using the Illumina HumanOmni1-Quad_v1 BeadChip system. Genotypes were called using the Illumina GenomeStudio software.

**CROATIA-Vis:** The CROATIA-Vis study genotyping used the Illumina HAP300v1 SNP chip. Genotype quality control excluded SNPs with a call rate <95%, MAF <0.01, HWE p<10e-6. Analysis was performed using GenABEL with first 3 principal components accounting for population stratification and “mmscore“ option to account for relationships. SNPs were imputed to HapMap22, build 36, using MACHv1.16. and GenABEL derived residuals were analysed using ProbABEL.

**FHS:** Genotyping was carried out as a part of the SNP Health Association Resource project using the Affymetrix 500K mapping array (250K Nsp and 250K Sty arrays) and the Affymetrix 50K supplemental gene focused array on 9274 individuals. Genotyping resulted in 503 551 SNPs with successful call rate > 95% and HWE P >1E-6 on 8481 individuals with call rate > 97%. Imputation of ~2.5 million autosomal SNPs in HapMap with reference to release 22 CEU sample was conducted using the algorithm implemented in MACH. The final population for fibrinogen analysis included 7022 individuals (original cohort, n=383; offspring, n=2,806; third generation, n=3,833).

**HBCS:** DNA was extracted from blood samples and genotyping was performed with the modified Illumina 610K chip by the Wellcome Trust Sanger Institute, Cambridge, UK according to standard protocols. Genomic coverage was extended by imputation using the HapMap phase II CEU data as the reference sample and MACH software. Association analyses were conducted with Quicktest (directly genotyped SNPs) and ProbABEL (imputed SNPs) by using robust variance estimates.

**InCHIANTI:** Illumina Infinium HumanHap 550K SNP arrays were used for genotyping [14].

**KORA F3 and KORA F4:** Genotyping for F3 was performed using Affymetrix 500K Array Set consisting of two chips (Sty I and Nsp I). The F4 samples were genotyped with the Affymetrix Human SNP Array 6.0. Hybridisation of genomic DNA was done in accordance with the manufacturer’s standard recommendations. Genotypes were determined using BRLMM clustering algorithm (Affymetrix 500K Array Set) or Birdseed2 clustering algorithm (Affymetrix Array 6.0). For quality control purposes, we applied a positive control and a negative control DNA every 48 samples (F3) or 96 samples (F4). On chip level only subjects with overall genotyping efficiencies of at least 93% were included. In addition the called gender had to agree with the gender in the MONICA/KORA study database. Imputation of genotypes was performed using maximum likelihood method with the software MACH v1.0.9 (F3) and MACH v1.0.15 (F4).

**LBC1921 and LBC1936:** A detailed description of the genotyping method is available elsewhere [38]. In brief, genotyping was performed on Illumina Human 610-Quadv1 chip on blood-extracted DNA. Standard quality control measures were applied including the following thresholds: Sample call rate ≥ 0.95, SNP call rate ≥ 0.98, minor allele frequency ≥ 0.01, and Hardy-Weinberg Equilibrium test with P ≥ 0.001. Individuals were also removed due to gender discrepancies, relatedness (proportion of IBD > 0.25) and evidence of non-European descent. Approximately 2.5M common SNPs included in HapMap, using the HapMap phase II CEU data as the reference sample were imputed. NCBI build 36 (UCSC hg18) was used and genotype data were imputed using MACH software. Linear regression analysis for an additive genetic model was performed using ProbABEL software, incorporating dosage information and including age and sex as covariates in the model.

**MARTHA:** The MARTHA study sample was typed with the Illumina Human610-Quad Beadchip. SNPs showing significant (P < 10-5) deviation from Hardy-Weinberg equilibrium, with minor allele frequency (MAF) less than 1% or genotyping call rate <99%, in each study were filtered out. After the filtering, 494,721 and 501,773 autosomal SNPs were left for association analysis and further used for imputing ~2.5 million autosomal SNPs according to the CEU HapMap release 21 reference dataset. The imputation was performed using MACH v1.0.16.

Individuals with genotyping success rates less than 95% were excluded from the analyses, as well as individuals demonstrating close relatedness as detected by pairwise clustering of identity by state distances (IBS) and multi-dimensional scaling (MDS) implemented in PLINK software [39]. Non-European ancestry was also investigated using the Eigenstrat program [35].

**NTR:** Three platforms were used to genotype the data: AFFY/Perlegen 660K, Illumina 370K and Illumina 660K. Per platform the quality control inclusion thresholds for SNPs were MAF > 1%, HWE > 0.00001, call rate > 95% and 0.30 < Heterozygosity < 0.35. Samples were excluded from the data if their expected sex and IBD status did not match, or if the genotype missing rate was above 10%. For each platform all SNPs were aligned to the positive strand of the Hapmap 2 Build 36 release 24 CEU reference set. The alignment was checked using individuals and family members tested on multiple platforms. SNPs were excluded per platform if allele frequencies differed more than 15% with the reference set and/or the other platforms. The data of the three chips were then imputed with the IMPUTE program on Hapmap 2 build 36rel24 (J. Marchini). From the imputed sets, SNPs were removed if the MAF had a difference larger than 0.15 between subsets (same reference alleles). The remaining SNPs were merged into one single set. Afterwards, bad imputed SNPs were removed based on HWE < 0.00001, proper info < 0.40 and MAF < 1%.

**ORCADES:** The ORCADES study genotyping used the Illumina HAP300v2 SNP chip. Genotype quality control excluded SNPs with a call rate <98%, MAF <0.01, HWE p<10e-6. Analysis was performed using GenABEL with first 3 principal components accounting for population stratification and “mmscore“ option to account for relationships. SNPs were imputed to HapMap22, build 36, using MACHv1.16 and GenABEL derived residuals were analysed using ProbABEL.

**PROCARDIS:** PROCARDIS was carefully genotyped using Illumina Human 1M and 610K quad arrays on a total of 6000 patients with CAD and 7,500 control subjects. SNPs with low call rate (<95%), low minor allele frequency (MAF<0.01) or with HWE P-Values <1.0x10^-6^ were filtered for quality control purposes. After filtering, 498,717 (1M) and 414,950 (610K) variants were included for imputation. Imputation of genotypes was performed using MACH1.0.16 according to HapMap build 36.

**PROSPER:** A whole genome wide screening has been performed in the sequential PROSPER/PHASE project with the use of the Illumina 660K beadchip. Of 5763 subjects DNA was available for genotyping. Genotyping was performed with the Illumina 660K beadchip, after QC (call rate <95%) 5,244 subjects and 557,192 SNPs were left for analysis. These SNPs were imputed to 2.5 million SNPs based on the HAPMAP built 36 with MACH imputation software [40].

**RS:** Genotyping was conducted using the Illumina 550K array. SNPs were excluded for minor allele frequency ≤1%, Hardy-Weinberg equilibrium (HWE) p<10^-5^, or SNP call rate ≤90% resulting in data on 530,683 SNPs. Imputation was done with reference to HapMap release 22 CEU using the maximum likelihood method implemented in MACH.

**SardiNIA:** Genotyping started on 2004, and different subsets of samples have been genotyped with different SNP arrays. Specifically, 1,412 were genotyped with the 500K Affymetrix Mapping Array set; 3,329 with the 10K Mapping Array set, with 436 individuals genotyped with both arrays; 1,097 individuals with the 6.0 Affymetrix chip, of which 1,004 and 66 of those were typed with the 10K and 500K chips respectively. Quality controls filters for the 500K and 10K array have been previously described [41,42]. For the Affymetrix 6.0 chip, we removed SNPs with call rate <95%, MAF <1% and HWE p value<10-6. We also discarded SNPs that showed an excess of mendelian errors and SNPs in common with the 500K showing an excess of discordant genotypes (>3 over 66 samples).

After performing quality control checks and merging genotypes from the three gene chip platform, we used 731,209 QCed autosomal markers to estimates genotypes for all polymorphic SNPs in the CEU HapMap population [37,43] in the individuals genotyped with the 500K Array and the 6.0 Affymetrix chip separately, using the MaCH software. Taking advantage of the relatedness among individuals in the SardiNIA sample, we carried out a second round of computational analysis to impute genotypes at all SNPs in the individuals who were genotyped only with the Affymetrix Mapping 10K Array, being mostly offspring and siblings of the individuals genotyped at high density. At this second round of imputation, we focused on the SNPs for which the imputation procedure predicted r2>0.30 between true and imputed genotypes and for which the inferred genotype did not generate an excess of Mendelian Errors. The within-family imputation procedure is implemented in Merlin software [44,45]. Overall, a total of 2,325,980 autosomal SNPs were selected for GWAS. Due to computational constraints, we divided large pedigrees into sub-units with “bit-complexity” of 21 or less (typically, 25-30 individuals) before analysis. Samples with high missing rate and cryptic IBS were excluded from the analysis.

**SHIP:** The SHIP samples were genotyped using the Affymetrix Human SNP Array 6.0. Hybridisation of genomic DNA was done in accordance with the manufacturer's standard recommendations. The genetic data analysis workflow was created using the Software InforSense. Genetic data were stored using the database Caché (InterSystems). Genotypes were determined using the Birdseed2 clustering algorithm. For quality control purposes, several control samples where added. On the chip level, only subjects with a genotyping rate on QC probesets (QC callrate) of at least 86% were included. All remaining arrays had a sample callrate > 92%. The overall genotyping efficiency of the GWA was 98.55 %. Imputation of genotypes in SHIP was performed with the software IMPUTE v0.5.0 based on HapMap II CEU. Duplicate samples on basis by estimated IBD as well as samples with mismatch between reported and genotyped gender were excluded prior to the analysis. Genome-wide association tests were performed using QUICKTEST v0.94 (http://toby.freeshell.org/software/quicktest.shtml). Uncertainties for imputed genotypes were taken into account for association testing.

**WGHS:** Genotyping in the WGHS was performed using the Infinium II technology from Illumina (San Diego, CA, USA). Either the HumanHap300 Duo+ or the HumanHap300 Duo and I-select chips were used, with identical custom content on both chips for a total of 363,808 SNPs genotyped. SNPs with call rates <90% and all participant samples with >2% missing genotypes were excluded from further analysis. Genotype imputation was performed using MaCH 1.0.16 to HapMap release 22.

**Imputation methods**

In each cohort, imputation was performed for all SNPs passing the quality-control criteria. This approach imputed unmeasured genotypes based on phased haplotypes observed in the HapMap CEU sample. Imputation estimations were summarized as an allele dosage defined as the expected number of copies of the minor allele at that SNP (a continuous value between 0 and 2) for each genotype. For each SNP, a ratio of observed to expected variance (OEV) of the dosage statistic was estimated ranging from 0 to 1 (poor to excellent) and reflecting imputation quality.

**Statistical analysis**

In each study, linear regression analyses with untransformed fibrinogen as outcome variable were performed separately. Adjustments were made for age (linear) and sex, except WGHS which included only women and adjusted only for age. Additionally, several studies adjusted further for specific covariates: ARIC adjusted additionally for center and B58C adjusted additionally for laboratory batch, time of day, month of examination, and postal delay. Moreover, to account for potential population stratification, further adjustment for principal components, multidimensional scaling or country was done if required in CROATIA-Vis, MARTHA, ORCADES, PROCARDIS and PROSPER. Family structure was taken into account in CROATIA-VIS, FHS, ORCADES and PROCARDIS. In FHS, a linear mixed effects model was used with a fixed additive effect for the SNP genotype, fixed covariate effects, random family-specific additive residual polygenic effects to account for within family correlations, and a random environment effect. Subjects on anticoagulant therapy at the time of examination were excluded in B58C, MARTHA and PROCARDIS from all analyses.

**Supporting Information References**

1. The ARIC investigators (1989) The Atherosclerosis Risk in Communities (ARIC) Study: design and objectives. Am J Epidemiol 129: 687-702.

2. Strachan DP, Rudnicka AR, Power C, Shepherd P, Fuller E, et al. (2007) Lifecourse influences on health among British adults: effects of region of residence in childhood and adulthood. Int J Epidemiol 36: 522-531.

3. The Wellcome Trust Case Control Consortium (2007) Genome-wide association study of 14,000 cases of seven common diseases and 3,000 shared controls. Nature 447: 661-678.

4. Barrett JC, Clayton DG, Concannon P, Akolkar B, Cooper JD, et al. (2009) Genome-wide association study and meta-analysis find that over 40 loci affect risk of type 1 diabetes. Nat Genet 41: 703-707.

5. Moffatt MF, Gut IG, Demenais F, Strachan DP, Bouzigon E, et al. (2010) A large-scale, consortium-based genomewide association study of asthma. N Engl J Med 363: 1211-1221.

6. Friedman GD, Cutter GR, Donahue RP, Hughes GH, Hulley SB, et al. (1988) CARDIA: study design, recruitment, and some characteristics of the examined subjects. J Clin Epidemiol 41: 1105-1116.

7. Fried LP, Borhani NO, Enright P, Furberg CD, Gardin JM, et al. (1991) The Cardiovascular Health Study: design and rationale. Ann Epidemiol 1: 263-276.

8. Vitart V, Rudan I, Hayward C, Gray NK, Floyd J, et al. (2008) SLC2A9 is a newly identified urate transporter influencing serum urate concentration, urate excretion and gout. Nat Genet 40: 437-442.

9. Feinleib M, Kannel WB, Garrison RJ, McNamara PM, Castelli WP (1975) The Framingham Offspring Study. Design and preliminary data. Prev Med 4: 518-525.

10. Barker DJ, Osmond C, Forsen TJ, Kajantie E, Eriksson JG (2005) Trajectories of growth among children who have coronary events as adults. N Engl J Med 353: 1802-1809.

11. Eriksson JG, Osmond C, Kajantie E, Forsen TJ, Barker DJ (2006) Patterns of growth among children who later develop type 2 diabetes or its risk factors. Diabetologia 49: 2853-2858.

12. Rikkonen K, Pesonen AK, Heinonen K, Lahti J, Kajantie E, et al. (2008) Infant growth and hostility in adult life. Psychosom Med 70: 306-313.

13. Ferrucci L, Bandinelli S, Benvenuti E, Di Iorio A, Macchi C, et al. (2000) Subsystems contributing to the decline in ability to walk: bridging the gap between epidemiology and geriatric practice in the InCHIANTI study. J Am Geriatr Soc 48: 1618-1625.

14. Melzer D, Perry JR, Hernandez D, Corsi AM, Stevens K, et al. (2008) A genome-wide association study identifies protein quantitative trait loci (pQTLs). PLoS Genet 4: e1000072.

15. Tanaka T, Shen J, Abecasis GR, Kisialiou A, Ordovas JM, et al. (2009) Genome-wide association study of plasma polyunsaturated fatty acids in the InCHIANTI Study. PLoS Genet 5: e1000338.

16. Holle R, Happich M, Lowel H, Wichmann HE (2005) KORA--a research platform for population based health research. Gesundheitswesen 67 Suppl 1: S19-S25.

17. Wichmann HE, Gieger C, Illig T (2005) KORA-gen--resource for population genetics, controls and a broad spectrum of disease phenotypes. Gesundheitswesen 67 Suppl 1: S26-S30.

18. Deary IJ, Whiteman MC, Starr JM, Whalley LJ, Fox HC (2004) The impact of childhood intelligence on later life: following up the Scottish mental surveys of 1932 and 1947. J Pers Soc Psychol 86: 130-147.

19. Deary IJ, Gow AJ, Taylor MD, Corley J, Brett C, et al. (2007) The Lothian Birth Cohort 1936: a study to examine influences on cognitive ageing from age 11 to age 70 and beyond. BMC Geriatr 7: 28.

20. Antoni G, Oudot-Mellakh T, Dimitromanolakis A, Germain M, Cohen W, et al. (2011) Combined analysis of three genome-wide association studies on vWF and FVIII plasma levels. BMC Med Genet 12: 102.

21. Germain M, Saut N, Greliche N, Dina C, Lambert JC, et al. (2011) Genetics of venous thrombosis: insights from a new genome wide association study. PLoS One 6: e25581.

22. Willemsen G, de Geus EJ, Bartels M, van Beijsterveldt CE, Brooks AI, et al. (2010) The Netherlands Twin Register biobank: a resource for genetic epidemiological studies. Twin Res Hum Genet 13: 231-245.

23. McQuillan R, Leutenegger AL, Abdel-Rahman R, Franklin CS, Pericic M, et al. (2008) Runs of homozygosity in European populations. Am J Hum Genet 83: 359-372.

24. Clarke R, Peden JF, Hopewell JC, Kyriakou T, Goel A, et al. (2009) Genetic variants associated with Lp(a) lipoprotein level and coronary disease. N Engl J Med 361: 2518-2528.

25. Shepherd J, Blauw GJ, Murphy MB, Cobbe SM, Bollen EL, et al. (1999) The design of a prospective study of Pravastatin in the Elderly at Risk (PROSPER). PROSPER Study Group. PROspective Study of Pravastatin in the Elderly at Risk. Am J Cardiol 84: 1192-1197.

26. Shepherd J, Blauw GJ, Murphy MB, Bollen EL, Buckley BM, et al. (2002) Pravastatin in elderly individuals at risk of vascular disease (PROSPER): a randomised controlled trial. Lancet 360: 1623-1630.

27. Hofman A, van Duijn CM, Franco OH, Ikram MA, Janssen HL, et al. (2011) The Rotterdam Study: 2012 objectives and design update. Eur J Epidemiol 26: 657-686.

28. Pilia G, Chen WM, Scuteri A, Orru M, Albai G, et al. (2006) Heritability of cardiovascular and personality traits in 6,148 Sardinians. PLoS Genet 2: e132.

29. Volzke H, Alte D, Schmidt CO, Radke D, Lorbeer R, et al. (2011) Cohort profile: the study of health in Pomerania. Int J Epidemiol 40: 294-307.

30. Ridker PM, Chasman DI, Zee RY, Parker A, Rose L, et al. (2008) Rationale, design, and methodology of the Women's Genome Health Study: a genome-wide association study of more than 25,000 initially healthy american women. Clin Chem 54: 249-255.

31. CLAUSS A (1957) [Rapid physiological coagulation method in determination of fibrinogen]. Acta Haematol 17: 237-246.

32. Rudnicka AR, Rumley A, Lowe GD, Strachan DP (2007) Diurnal, seasonal, and blood-processing patterns in levels of circulating fibrinogen, fibrin D-dimer, C-reactive protein, tissue plasminogen activator, and von Willebrand factor in a 45-year-old population. Circulation 115: 996-1003.

33. Cushman M, Cornell ES, Howard PR, Bovill EG, Tracy RP (1995) Laboratory methods and quality assurance in the Cardiovascular Health Study. Clin Chem 41: 264-270.

34. Kannel WB, Wolf PA, Castelli WP, D'Agostino RB (1987) Fibrinogen and risk of cardiovascular disease. The Framingham Study. JAMA 258: 1183-1186.

35. Price AL, Patterson NJ, Plenge RM, Weinblatt ME, Shadick NA, et al. (2006) Principal components analysis corrects for stratification in genome-wide association studies. Nat Genet 38: 904-909.

36. Li Y, Willer C, Sanna S, Abecasis G (2009) Genotype imputation. Annu Rev Genomics Hum Genet 10: 387-406.

37. Li Y, Willer CJ, Ding J, Scheet P, Abecasis GR (2010) MaCH: using sequence and genotype data to estimate haplotypes and unobserved genotypes. Genet Epidemiol 34: 816-834.

38. Houlihan LM, Davies G, Tenesa A, Harris SE, Luciano M, et al. (2010) Common variants of large effect in F12, KNG1, and HRG are associated with activated partial thromboplastin time. Am J Hum Genet 86: 626-631.

39. Purcell S, Neale B, Todd-Brown K, Thomas L, Ferreira MA, et al. (2007) PLINK: a tool set for whole-genome association and population-based linkage analyses. Am J Hum Genet 81: 559-575.

40. Trompet S, de Craen AJ, Postmus I, Ford I, Sattar N, et al. (2011) Replication of LDL GWAs hits in PROSPER/PHASE as validation for future (pharmaco)genetic analyses. BMC Med Genet 12: 131.

41. Scuteri A, Sanna S, Chen WM, Uda M, Albai G, et al. (2007) Genome-wide association scan shows genetic variants in the FTO gene are associated with obesity-related traits. PLoS Genet 3: e115.

42. Sanna S, Jackson AU, Nagaraja R, Willer CJ, Chen WM, et al. (2008) Common variants in the GDF5-UQCC region are associated with variation in human height. Nat Genet 40: 198-203.

43. Marchini J, Howie B, Myers S, McVean G, Donnelly P (2007) A new multipoint method for genome-wide association studies by imputation of genotypes. Nat Genet 39: 906-913.

44. Abecasis GR, Cherny SS, Cookson WO, Cardon LR (2002) Merlin--rapid analysis of dense genetic maps using sparse gene flow trees. Nat Genet 30: 97-101.

45. Chen WM, Abecasis GR (2007) Family-based association tests for genomewide association scans. Am J Hum Genet 81: 913-926.
